# Salicylic acid functionalized zein for improving plant stress resistance and as a nanopesticide carrier with enhanced anti-photolysis ability

**DOI:** 10.1186/s12951-023-01777-7

**Published:** 2023-01-20

**Authors:** Haozhao Yan, Li Hao, Huayao Chen, Xinhua Zhou, Hongbing Ji, Hongjun Zhou

**Affiliations:** 1 Innovative Institute for Plant Health, School of Chemistry and Chemical Engineering, Zhongkai University of Agriculture and Engineering, Guangzhou, 510225 Guangdong People’s Republic of China; 2 Fine Chemical Industry Research Institute, School of Chemistry, Sun Yat-Sen University, Guangzhou, Guangdong People’s Republic of China; 3 Key Laboratory of Agricultural Green Fine Chemicals of Guangdong Higher Education Institution, Zhongkai University of Agriculture and Engineering, Guangzhou, 510225 Guangdong People’s Republic of China; 4 Key Laboratory of Green Prevention and Control on Fruits and Vegetables in South China, Ministry of Agriculture and Rural Affairs, Chengdu, People’s Republic of China

**Keywords:** Zein, Nanoparticle, Cytotoxicity, Transfer factor, Salt stress

## Abstract

**Background:**

There is a serious global problem of salinization of arable land, causing large reduction in world food production. Use of plant hormones is an effective way to reduce damage caused to crops and salt stress.

**Results:**

In this study, PEI-EDA was modified with AM-zein and grafted with plant hormone SA (AM-zein-SA) and used as a nano-pesticide carrier to load emamectin benzoate (EB). The use of AM-zein-SA as a nano-pesticide carrier could reduce the damage caused by salt stress to crops. The structure of AM-zein-SA was characterized by FTIR, UV, fluorescence, Raman, and ^1^H NMR spectroscopic techniques. AM-zein-SA could effectively improve the resistance of EB to ultraviolet radiations, resistance of cucumber to salt stress, and the absorption of EB by plants. The experimental results showed that AM-zein-SA could effectively improve the anti-UV property of EB by 0.88 fold. When treated with 120 mmol NaCl, the germination rate of cucumber seeds under salt stress increased by 0.93 fold in presence of 6.25 mg/L carrier concentration. The POD and SOD activities increased by 0.50 and 1.21 fold, whereas the content of MDA decreased by 0.23 fold. In conclusion, AM-zein-SA nano-pesticide carrier could be used to improve the salt resistance of crops and the adhesion of pesticides to leaves.

**Conclusion:**

AM-zein-SA, without undergoing any changes in its insecticidal activity, could simultaneously improve the salt stress resistance and salt stress germination rate of cucumber, reduce growth inhibition due to stress under high-concentration salt, and had a good effect on crops. In addition, EB@AM-zein-SA obviously improved the upward transmission rate of EB, as compared with EB. In this study, SA was grafted onto zein-based nano-pesticide carrier, which provided a green strategy to control plant diseases, insects, and pests while reducing salt stress on crops in saline-alkali soil.

**Graphical Abstract:**

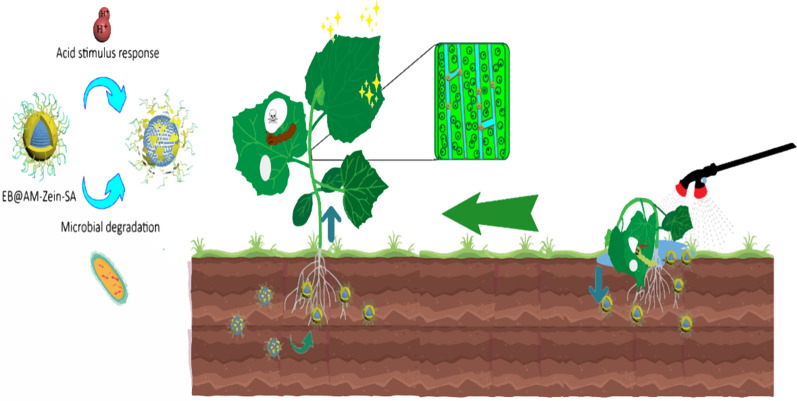

## Introduction

With world-wide increase in crop produce, the use of pesticides has also increased. Annually, the use of pesticides globally is as much as 3 billion kilograms [1]. Also, about 90% of the pesticides used, fail to reach the target site and are lost to the environment [2]. Therefore, pesticides are being excessively used to ensure good agricultural production, which cause serious environmental pollution and ultimately pose risks to human health [3]. To tackle the problem of environmental pollution caused by excessive use of pesticides, researchers have developed slow-release pesticides and controlled-release technologies, which are aimed at increasing the life-cycle of agricultural chemicals, reduce environmental pollution, and also to reduce the use of agricultural chemicals. Several kinds of materials are being used presently for the slow release of agricultural chemicals, which are as follows: (1) Metal nanoparticles, (2) Metal oxide nanoparticles, (3) Porous nanoparticles, (4) Micelles, and (5) Liposomes [4]. Among them, chitosan, sodium alginate, cellulose, and zein are micelle carriers based on natural polymers, with good biocompatibility.

Zein has low nutritional value, but is widely used in medicine and biomedicine owing to its biocompatibility. The current applications of zein in agriculture include improvement of the anti-photolysis ability of some photosensitive pesticides, improvement of adhesivity of pesticides to leaves, development of environmentally friendly water-based pesticides, and increasing the utilization rate of pesticide [5, 6]. Currently, there are four main methods of using zein to load pesticides, which include anti-solvent precipitation, electro-spray, pH-driven self-assembly, and supercritical anti-solvent technology [7, 8]. The most simple and convenient method is the anti-solvent precipitation method, but since the isoelectric pH of zein is 6.2–6.8, the zein nanoparticles lose their original function due to the aggregation of zein. The isoelectric point of agglomeration of zein can be improved by addition of surfactants. In addition, the isoelectric point can also be chemically modified to prevent agglomeration of zein. The main principle is to increase the electrostatic repulsions and steric hindrance between nanoparticles through electrostatic interactions, hydrogen bonding, and hydrophobic interactions. Chen et al. [9] modified zein with ethylenediamine polyethylenimine. Zein modified by PEI-EDA showed changes in the ratio of positive and negative charges on the surface of zein nanoparticles. The increase in isoelectric point of zein resulted in stable dispersion of AM-zein nanoparticles under neutral conditions.

Biotic and abiotic stresses are the main factors responsible for reduction in crop yield. The main biotic stresses are caused due to phytophagous pests, disease-causing bacteria, and viruses [10]. The most common causes for abiotic stresses include salt, cold, heavy metal poisoning, drought, high temperature, and other major environmental factors. These stresses can lead to large reduction in crop yields and cause serious economic losses [11].

The salinization of cultivated land is one of the most serious problems affecting agricultural produce. The salinization area of cultivated land accounts for 20% of the total cultivated land area world-wide, resulting in 20% reduction of world food produce each year [12]. The cultivation of grains, such as rice, maize, wheat, and sorghum, which constitute the staple food of the world population, accounts for about 66% of the world's arable land. Therefore, the effects of salinization-alkalization of cultivated land are especially serious in terms of growth and yield, especially in case of rice, which is a salt-sensitive crop. Understanding the changes of physiological and biochemical indexes in the process of salt stress can improve the salt stress resistance of plants. The main reason of salt stress damage to plants is the increase of ROS, programmed cell death, DNA damage, lipid peroxidation, protein peroxidation, antioxidative metabolism imbalance, inhibition of electron transport system. Therefore, reducing the oxidation stress caused by ROS is the key to improve the salt tolerance of plants. Numerous studies have shown how plants affect ROS levels in enzymatic and non-enzymatic components, such as the following antioxidant enzymes, superoxide dismutase, catalase, peroxidase, Glutathione peroxidase, ascorbate peroxidase, Glutathione S-transferases, Glutathione reductase, which are closely related to the breakdown of ROS. On the other hand, plant antioxidant enzyme systems are composed of salicylic acid, methyl jasmonate, ethylene, jasmonic acid, abscisic acid, gibberellin and other plant hormones together constitute the activation of antioxidant enzymes involved in the pathway of synthesis [13–15]. At present, the main methods for solving the problem of reduced grain yield caused by soil salinization and their limitations are as follows: (1) Improvement of soil, but the improvement of salinized land requires a great deal of time and efforts and long-term maintenance of the improved land [16], due to its distribution world over. (2) Genetically modified salt-tolerant genotypic crops can help in greatly overcoming salt and alkali resistance, but the biological safety of transgenic crops needs long time verification [17, 18]. (3) Spraying with plant hormone can reduce crop-death and improve the yields, as a response to salt stress. However, there is a lag in the application of plant hormone, thus seriously affecting its applications. Moreover, the persistence of phytohormone treatment is poor, requiring 5–7 days for supplementation and the artificial cost is relatively high [19, 20].

Salicylic acid (SA) is an endogenous hormone present in plants. SA can regulate various physiological and biochemical processes in plants to relieve stress, when they are exposed to adverse environmental conditions (such as salt injury, water shortage, cold injury, heavy metal pollution.) [21, 22]. For example, the cold tolerance of maize seedlings was improved by the application of exogenous salicylic acid [23] and the toxicity of *Chenopodium album* was regulated by pretreatment with exogenous SA [24]. The main reason for improvement of tolerance to plant stress by SA is that it can increase the activity of antioxidant enzymes, such as peroxidase (POD) and superoxide dismutase (SOD) to rapidly decompose and produce reactive oxygen species (ROS) in plant cells, when plants face adverse conditions. The production of a large number of ROS leads to an attack on the cell membrane, resulting in the leakage of cell contents, and ultimately causing cell death [25].

However, the main limitations of application of salicylic acid are as follows: (1) excessive use of plant hormones causes’ plant damage, (2) plant hormone use persistence is poor. Hadi et al. [26] treated barley at grain filling stage with SA under salt stress. With increase in SA concentration, the carbohydrate and starch contents in barley grain increased initially and then decreased. Mohammadi et al. [27] treated salt-stressed quinoa with 0.75 mM and 1.5 mM concentrations of SA and found that the growth of quinoa was promoted at low concentrations. However, there was no significant increase or negative effect at higher concentration. These results showed that high concentration of SA had a negative effect on carbohydrate accumulation in barley grain during grain filling.

**Fig. 1:**
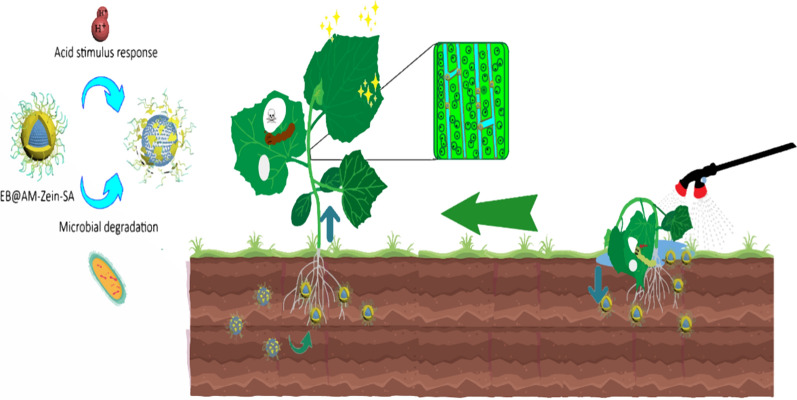
Spraying of EB@AM-zein-SA on cucumber plants under salt stress enhanced salt tolerance, drug release property, and uptake of nanoparticles

To reduce the damage caused due to salt stress on crops and improve the efficiency of pesticide transportation in crops, we proposed to graft the plant hormone, SA, on AM-zein, a nano-pesticide carrier. The structure of AM-zein-SA was characterized by Fourier transform infrared spectroscopy, ultraviolet spectroscopy, fluorescence spectroscopy, Raman microscopy, scanning electron microscopy, and ^1^H NMR spectroscopy. The performance of EB@AM-zein-SA as a nanopesticide was evaluated in terms of foliar affinity, storage stability, insecticidal activity, UV resistance, and pH-responsive sustained release. The effects of the nano-pesticide carrier, AM-zein-SA, on the germination of cucumber seeds under salt stress and the changes in activities of antioxidant enzymes were also studied. The absorptivities and conduction properties of nano-pesticide particles in plants were investigated. Figure 1 is a schematic diagram showing that spraying of EB@AM-zein-SA on cucumber plants under salt stress improved the salt tolerance, drug release property, and uptake of nanoparticles.

## Materials and methods

### Plant

Cucumber seeds (*Cucumis* *sativus*, Zhongnong No.6, China) were sown in perlite. Cucumber plants with different number of true leaves were used for the tests, according to test requirements. All the tested leaves had the same growth characteristics. The cucumber seeds were treated with 10% H_2_O_2_ for 30 min and then transferred into 500 mL deionized water and incubated at 25 °C for 8 h. The seeds floating on the surface were removed. Then it will be used in the germination experiment of cucumber seeds under salt stress, in which a total of 1800 cucumber seeds were used in the germination experiment under salt stress.

### Experimental materials

Zein (purity 91%), L-leucine, thiobarbituric acid, and sodium hydroxide were purchased from Macklin (Shanghai, China). Ethylene glycol diglycidyl ether (EGDE), PEI-EDA (average molecular weight ~ 800), phthalaldehyde, 2-mercaptoethanol, 1-ethyl-(3-dimethylaminopropyl)carbodiimide hydrochloride (EDC), N-hydroxysuccinimide (NHS), guaiacol, hydrogen peroxide (30%), riboflavin, disodium ethylenediaminetetraacetate (EDTA-Na_2_), D-methionine, nitroblue tetrazolium (NBT) (95%), sodium dihydrogen phosphate dihydrate, disodium hydrogen phosphate heptahydrate, sodium dodecyl sulfonate (electrophoresis level), and sodium tetraborate decahydrate were all obtained from Aladdin (Shanghai, China). Potassium bromide was supplied by Guangzhou Chemical Reagent Factory (Guangzhou, China). Standard protein (Marker), electrophoresis buffer, Coomassie brilliant blue fast staining solution, and 30% acrylamide were all procured from Solaibio (Beijing China). Ethanol, trichloroacetic acid, salicylic acid, and concentrated hydrochloric acid were provided by Tianjin Damao Chemical Reagent Factory (Tianjin, China). All chemicals were analytically pure, except for specific reagents, and they were used without further purification.

### Preparation of salicylic acid grafted zein(AM-zein-SA)

Zein (1 g) and ethylene glycol diglycidyl ether (EDGE, 0.5 g) were dissolved in 50 mL of 70% ethanol–water (v/v) solution and stirred at room temperature for 6 h. Subsequently, ethylenediamine-terminated polyethyleneimine (PEI-EDA, 3 g) was added and the reaction mixture was continuously stirred for 1 h and then transferred to a dialysis bag with a molecular weight cut-off of 5000 Da for 24 h to remove the unreacted reactants, which was followed by freeze-drying to obtain AM-zein [9].

**Fig. 2:**
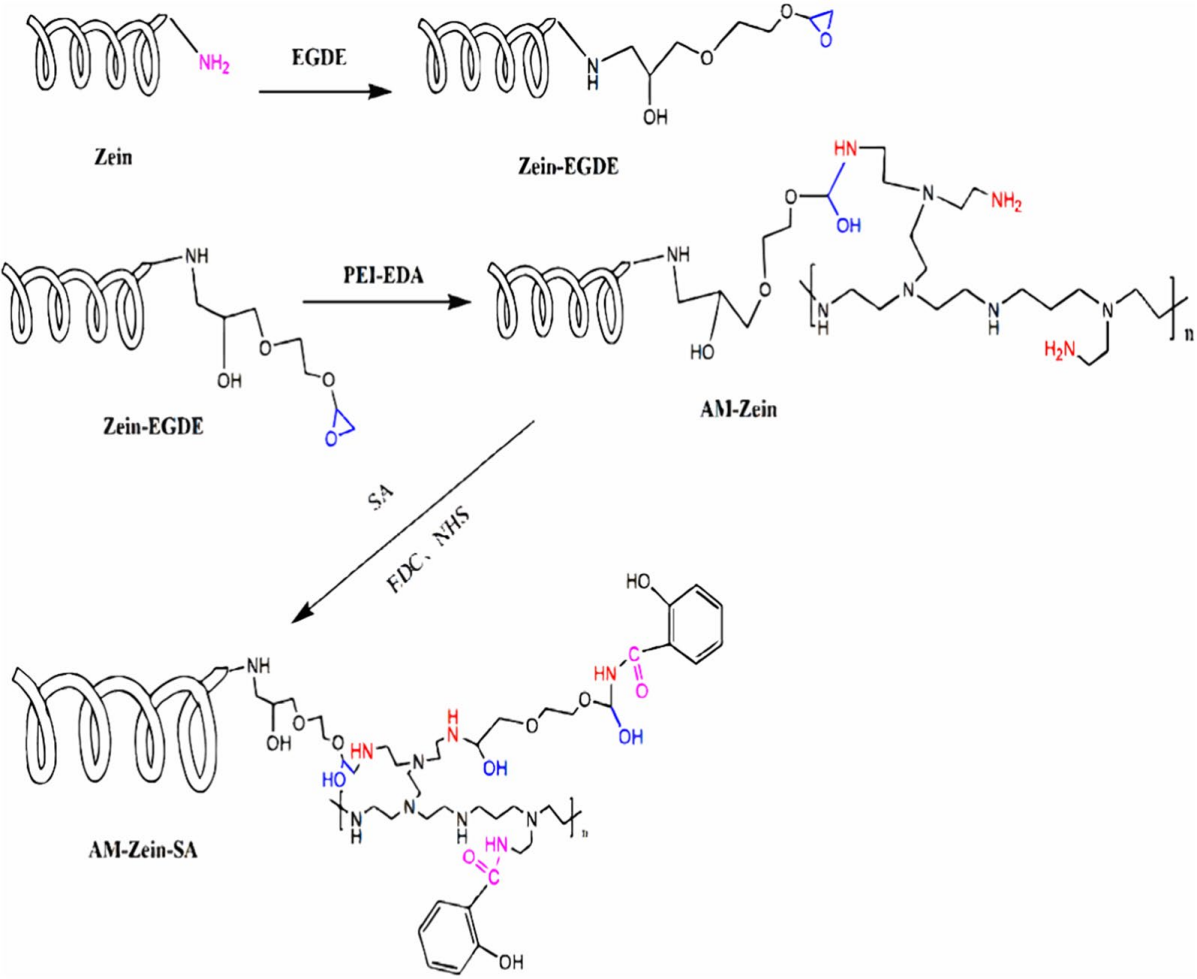
The proposed route for the synthesis of AM-zein-SA

SA (0.5 g), EDC (0. 3 g), and NHS (0.2 g) were dissolved in 50 mL of 70% (v/v) ethanol–water solution. Then, the pH was adjusted to 5.5 using 0.1 mol/L sodium hydroxide aqueous solution. SA was activated by stirring for 2 h at room temperature. Subsequently, AM-zein (0.5 g) was added to SA solution and the pH was adjusted to 5.5 using 0.1 mol/L hydrochloric acid solution with continuous stirring. The mixture was reacted for 24 h at room temperature. The product was dialyzed for 1 d in a dialysis bag with a molecular weight cut-off of 5000 Da and then freeze-dried to obtain the sample of salicylic acid grafted AM-zein. The sample was named AM-zein-SA. Figure 2 shows the proposed route for the synthesis of AM-zein-SA.

### Preparation of EB@AM-zein-SA and EB@AM-zein

EB@AM-zein was prepared by an improvised anti-solvent method. AM-zein-SA (100 mg), AM-zein (100 mg), and EB (20 mg) were dissolved in 5 mL of 70% (v/v) ethanol–water solution and ultrasonicated for 10 min. Subsequently, the solution mixture of EB and AM-zein was quickly poured into 95 mL of deionized water at pH 3 and stirred magnetically to obtain EB@AM-zein. Finally, the pH values of EB@AM-zein dispersions were adjusted to 7 using 0.1 mol/L sodium hydroxide solution. The preparation of EB@AM-zein-SA was also carried out by the same above methodology. The samples were freeze-dried for further characterization.

### Characterization of the carrier structure

The structures of zein, AM-zein, AM-zein-SA, and SA were all analyzed by DXR2 Raman spectroscopy (Thermo Fisher, USA). The zein, AM-zein, AM-zein-SA, and SA samples were ground into fine powders in a mortar and small amounts of sample powders were picked up by a capillary and placed on a glass slide. The objective lens was 10×, the incident wavelength was 532 nm, and the laser energy was 4 mW. The pinhole was set to 25 μm.

The structures of the samples were characterized by ^1^H NMR spectroscopy on a Bruker 400 M Proton NMR (Bruker, German). Accurately weighed 15 mg of AM-zein, AM-zein-SA, and SA samples were analyzed in deuterated-DMSO solvent.

The FTIR spectra of SA, zein, AM-zein, and AM-zein-SA samples were acquired on a Spectrum 100 Fourier transform infrared (FTIR) spectrometer (Perkin Elmer, USA). Each sample in the form of KBr pellet was scanned 32 times in the spectral range of 4000–400 cm^−1^.

The fluorescence absorption spectra of AM-zein, AM-zein-SA, SA, and zein were obtained using a LS45 fluorescence photometer (Perkin Elmer, USA). The SA, zein, AM-zein, and AM-zein-SA samples were prepared in 70% (v/v) ethanol–water solvent. The concentrations of the above sample solutions were 1 mg/mL and the incident wavelength was adjusted to 310 nm. The fluorescence spectra of the samples diluted to 1000 times were measured.

The morphologies of the samples were studied using a Sigma 300 scanning electron microscope (Zeiss, German). The samples were dried naturally after their syntheses, then sprayed with Pt for 15 min, and scanned at an accelerated voltage of 3 kV.

The free amino content in AM-zein was determined by OPA method [28]. The sample (50 mg) was dissolved in 5 mL of 70% (v/v) ethanol–water solution and 1 mL of 15% (w/v) trichloroacetic acid was added to the solution over a period of 10 min at 35 °C. A 200 μL of the supernatant was centrifuged and mixed with 4 mL OPA reagent in the same test tube for 5 min. The absorbance of the solution was determined using 3600-plus UV–visible near-infrared spectrophotometer (Shimadzu, Japan) at 340 nm. The amino content in the protein was calculated according from the standard curve of leucine (*A* = 0.30669*C*–0.01180, *R*^2^ = 0.9999).

The UV absorbances were characterized by a UV spectrophotometer. The absorbances of 100 mg/L solutions of zein, AM-zein, AM-zein-SA, and SA in 70% (v/v) ethanol–water solvent were recorded. 70% (v/v) ethanol–water solution was used as the blank background.

The molecular weight distributions of zein, AM-zein, and AM-zein-SA were determined by SDS-PAGE electrophoresis. Zein, AM-zein, and AM-zein-SA were dissolved in 70% (v/v) ethanol–water solutions to obtain 20 mg/mL solution concentrations. 1% SDS solution was added to 1 mL of each protein solution to obtain 2 mg/mL protein solution. Next, 80 uL protein solution 2 mg/mL was mixed with 20 uL, 5 × SDS–PAGE buffer solution to form the sample. After shaking, the mixtures were kept in a water bath at 100 ℃ for 10 min and the gels were then centrifuged for 5 min at 3000 rpm. The concentrations of acrylamide gel and concentrated gel were 15% and 5%, respectively, and a voltage of 120 V was setas, at which the gel was separated. When the sample reached the concentrated gel through the separating gel, the voltage was adjusted to 80 V. After electrophoresis, the gel was separated from the electrophoresis plate and stained with Coomassie brilliant blue fast staining solution.

### Stability of the nanoparticles

The pH of the freshly prepared EB@AM-zein and EB@AM-zein-SA dispersions were adjusted to the desired values (3, 5, 7, 9) using 0.1 mol/L hydrochloric acid solution or sodium hydroxide solution, and the effect of pH on their stabilities was studied.

Hydrodynamic diameter and Zeta potential were measured using a 90-plus laser particle size analyzer (Brookhaven, USA) to determine the stability of nanoparticles at different pH values. The storage stability of the nanoparticle also was evaluated at 26 °C and under neutral conditions for 28 days.

### Encapsulation efficiency and loading capacity

EB@AM-zein powder (10 mg) was dispersed in 4 mL of absolute ethanol. The dispersion was centrifuged at 12,000 rpm for 5 min. Then 1 mL of the sample solution was diluted to 10 mL in a volumetric flask and the concentration of the EB was assayed by ultraviolet spectroscopy at 245 nm. The free EB was calculated from the calibration curve of EB in ethanol (*A* = 33.34106C + 0.00841, *R*^2^ = 0.9999). All measurements were carried out in triplicates and their mean values were reported. The encapsulation efficiencies (*EE*) and loading capacities (*LC*) were calculated using formulae (1) and (2), respectively. According to the formulae (1) and (2), the *EE* and *LC* of EB in AM-zein and AM-zein-SA were 27.94 ± 0.08%, 4.66 ± 0.01% and 22.21 ± 0.05%, 3.70 ± 0.01% respectively.

1 $$EE = {{\left( {m_{{{\text{total}}\,{\text{EB}}}} - m_{{{\text{free}}\,{\text{EB}}}} } \right)} \mathord{\left/ {\vphantom {{\left( {m_{{{\text{total}}\,{\text{EB}}}} - m_{{{\text{free}}\,{\text{EB}}}} } \right)} {m_{{\,{\text{total}}\,{\text{EB}}}} \times \,100\% }}} \right. \kern-0pt} {m_{{\,{\text{total}}\,{\text{EB}}}} \times \,100\% }},$$

2 $$LC = {{\left( {m_{{{\text{total}}\,{\text{EB}}}} - m_{{{\text{free}}\,{\text{EB}}}} } \right)} \mathord{\left/ {\vphantom {{\left( {m_{{{\text{total}}\,{\text{EB}}}} - m_{{{\text{free}}\,{\text{EB}}}} } \right)} {m_{{{\text{EB}}@{\text{AM}} - {\text{zein}} - {\text{SA}}}} \times 100\% }}} \right. \kern-0pt} {m_{{{\text{EB}}@{\text{AM}} - {\text{zein}} - {\text{SA}}}} \times 100\% }},$$

where, *m*_total EB_ is the total mass of EB used for nanoparticles preparation, *m*_free EB_ is the weight of the unencapsulated EB, and *m*_EB@AM-zein-SA_ represents the total mass of the nanoparticles.

## In vitro release study

Freshly prepared 5 mL each of EB@AM-zein and EB@AM-zein-SA dispersions were taken in dialysis bags with a molecular weight cut-off of 5000 Da. The dialysis bags placed in the conical flasks containing the solutions 100 mL of 25% (v/v) ethanol–water solutions of pH 5, 7, and 9 were taken in brown conical flasks and placed on a rotating shaker at 30 °C.

During the release of EB, 1 mL of the solution was withdrawn from the conical flask at regular intervals and diluted in an volumetric flask to 10 mL. The brown Erlenmeyer flask was supplemented with the ethanol–water solution of the same pH each time. The absorbances of the diluted solutions were measured at a wavelength of 245 nm. The release amount of EB was calculated from the standard curve of absorbances of EB in aqueous ethanol. The cumulative release rate (*R*i) was calculated according to formula (3):

3 $$Ri = \left\{ {\begin{array}{*{20}l} {C_{i}^{*} 0.105/m_{EB} } &; {\left( {i = 1} \right)} \\ {C_{i}^{*} 0.105/m_{EB} + \sum\limits_{i = 1}^{i - 1} {C_{i}^{*} } 0.001/m_{EB} } &; {\left( {i1} \right)} \\ \end{array} } \right.,$$

where *C*_i_ is the concentration of EB in the buffer solution at different time periods (mg/L) and *m*_EB_ is the total mass of EB in the sample solution.

### Investigation of photodegradation

EB, EB@AM-zein, and EB@AM-zein-SA were diluted to concentrations of 100 mg/L. A 100 mL tube containing the sample solution was placed 5 cm away from the 300 W mercury lamp having ultraviolet wavelength of 365 nm and the magnetic stirrer was turned on to mix the solution well. 1 mL of the sample solution was withdrawn at regular intervals and diluted to 10 mL with anhydrous alcohol. The EB concentration was determined by LC-2030 plus high-performance liquid chromatography (HPLC, Shimadzu, Japan). The ratio of mobile phase was methanol: acetonitrile: ammonia water = 35:50:15, the flow rate was 1 mL/min, injection volume was 10 μL, column temperature was 40 °C, and the concentration of ammonia water was (v/v) 1:300. The chromatographic column used was Athena C18-WP, 100 Å, 4.6 mm × 150 mm, 5 μm, and the detection wavelength was 245 nm.

### Wettability test

The contact angle was measured using a Theta contact angle meter (Biolin, Sweden). Ignoring the thick veins of cucumber, the cucumber leaves were cut into strips and about 10 μL of sample solution was squeezed out through the micro-injector. The micro-injector was placed at a distance of 1 cm from the cucumber leaf. When the sample solution was squeezed out from the micro-injector and dropped on the cucumber leaf, the contact angle was measured.

### Leaf surface retention of EB preparation

The retention of pesticide drops on cucumber leaves was determined by the following method: The cucumber leaves were carefully cut into 2 × 2 cm^2^ pieces without damaging the main veins of cucumber leaves. The leaves of cucumber were soaked in EB (200 mg/L), EB@AM-zein (200 mg/L), and EB@AM-zein-SA (200 mg/L) solutions. After fully soaking, the leaves were vertically clamped with tweezers for 30 s until the drops stopped dripping from the leaves and they were weighed. The retention of droplets per unit area was calculated using formula (4):

4 $${\text{Retention}} = \left( {m_{{{\text{after}}}} - m_{{{\text{before}}}} } \right){/2}A,$$

where, *M*_after_ and *M*_before_ represented the weights of the leaf before and after soaking, respectively, and *A* represents the surface area of the leaf.

Retention of pesticides on leaf surface after washing by simulated rain was tested by adding 0.5 mL EB, EB@AM-zein, and EB@AM-zein-SA samples on each leaf. The leaves were dried naturally and washed at an angle of 45°, with 5 cm distance from the tap for 10 s, keeping the water flow constant. The leaves were then crushed by freezing under liquid nitrogen in a 10 mL centrifuge tube and dried in a ALPHA1-2 LD PLUS lyophilizer (German, Christ) for 1 day. The solution obtained by adding 5 mL of 90% acetonitrile in each tube for 20 min was collected and then centrifuged for 10 min at 12,000 rpm. 1 mL of the supernatant was injected into the sample bottle through a 220 nm filter and the EB content in the supernatant was determined by HPLC. The HPLC method was the same as that used for photodegradation investigation study. The samples in the CK group were dried without washing after adding 0.5 mL of EB, EB@AM-zein and EB@AM-zein-SA samples on each leaf, respectively.

### Seed germination under salt stress

A piece of filter paper was placed in each petri dish and sterilized in an autoclave. In each diameter of 6 cm petri dish were added 30 seeds and filled with 10 mL of respective nanocarrier solution. Six groups of parallel experiments were conducted. The number of geminating cucumber seeds in each culture dish was counted at 72 h and 120 h.

In Group A, AM-zein was mixed with 120 mmol NaCl solution. In Group B, AM-zein-SA was mixed with 120 mmol NaCl solution. Group 1–5 included 50, 25, 12.5, 6.25, 3.125 mg/L AM-zein or AM-zein-SA respectively. CK_1_ represented control group with deionized water, whereas CK_2_ represented 120 mmol NaCl solution.

### Determination of Peroxidase (POD) activity

The experimental method was slightly modified with respect to the principles and techniques of plant physiological and biochemical experiments [29]. CK_0_ was boiled for 5 min, whereas CK_1_, A, and B samples were treated in the same way as in "Seed germination under salt stress" section, whereas CK_2_ represented 60 mmol NaCl solution.

Cucumber seedlings at three-leaf stage were cultured in different solutions for 24 h. After 24 h of hydroponic cultivation, the cucumber leaves were plucked and then cut into thin strips and mixed evenly. The leaf strips (1 g) was taken in a mortar and to it was added 1 mL of 7.8 pH phosphate buffer solution of 0.05 mol/L concentration. Thereafter, the homogenate was transferred into a 5 mL centrifuge tube and 1 mL of buffer solution with phosphoric acid was added rinse the mortar and this solution was transferred into the centrifuge tube. The solution was centrifuged for 10 min at 3000 rpm and the supernatant was transferred to a 10 mL volumetric flask. The precipitates were extracted twice with 2 mL of phosphoric acid buffer and the supernatant was added into the volumetric flask and diluted 10 mL. The supernatant was preserved at 4 ℃.

In 10 mL centrifuge tube, phosphate buffer solution (2.9 mL, 0.05 mol/L), 2% H_2_O_2_ (1.0 mL), guaiacol (1.0 mL, 0.05 mol/L), and cucumber leaf extract POD solution (0.1 mL) were added sequentially and the reaction system was heated in a water bath at 37 °C for 2 min. Then, 5 mL phosphoric acid buffer was added to the 10 mL centrifuge tube and the absorbance of the solution was determined by ultraviolet spectroscopy at 470 nm within 5 min, at a rate of 0.01A A470 per minute, the peroxidase was 1 unit (U). The peroxidase activity was determined by formula (5).

5 $${\text{Peroxidase activity}}\left[ {{{\upmu } \mathord{\left/ {\vphantom {{\upmu } {\left( {{\text{g}} \cdot \min } \right)}}} \right. \kern-0pt} {\left( {{\text{g}} \cdot \min } \right)}}} \right] = \frac{{\Delta A470^{ * } VT}}{{m^{ * } Vs^{ * } 0.01^{ * } t}},$$

where Δ*A*470 represents change in absorbance with reaction time: *m* is the fresh weight (g) of cucumber leaves, *t* is the reaction time (min), *V*_T_ is the total volume of enzyme solution, and *V*_S_ is the volume of enzyme solution (mL).

### Determination of superoxide dismutase (SOD) activity

The method for determination of SOD activity refers to Bach-Pages et al. [30]. The method of configuration and grouping of the cucumber hydroponic solution was the same as that of POD test. Three real leaves of three-leaf-stage of cucumber were cut into strips after 24 h of cultivation in the above hydroponic medium. Pre-cooled phosphate buffer solution (pH = 7.8, 1 mL) was added to grind it into a homogenate in ice water bath. The homogenate was added into a 5 mL centrifuge tube, the mortar was rinsed with 2 mL phosphate buffer solution and mixed with the contents in the centrifuge tube and then centrifuged for 10 min at 4000 rpm. Phosphate buffer (0.05 mol/L, 1.5 mL), methionine (Met) solution (120 mmol/L, 0.3 mL), nitroblue tetrazolium solution (750 μmol/L, 0.3 mL), and EDTA-Na_2_ (100 μmol/L, 0.3 mL) were added to the finger-shaped tube in sequence, followed by riboflavin (20 μmol/L, 0.3 mL), enzyme 0.05 mL, and distilled water 0.25 mL. After mixing the two CK_0_ control tubes with phosphoric acid buffer instead of enzyme solution, one control tube was placed in dark place, while the other tubes were exposed to sunlight for 20 min at 4000 lx (the degree of exposure of each tube was the same, wherein the exposure time was shortened for high temperature and prolonged for low temperature). The absorbances of the other tubes were measured by ultraviolet spectroscopy at 560 nm.

The 50% inhibition of NBT photochemical reduction is an active unit (U) of SOD, which is calculated by formula (6).

6 $${\text{Total activity of SOD }}\left( {{\text{U}}/{\text{g}}} \right){ } = { }\frac{{{\text{(A}}_{{{\text{CK}}}} {\text{ - A}}_{{\text{E}}} {\text{)*V}}_{{\text{T}}} }}{{\frac{{1}}{{2}}{\text{*A}}_{{{\text{CK}}}} {\text{*m*V}}_{{\text{S}}} }},$$

where *A*_CK_ is the absorbance of the illumination control tube; *A*_E_ is the absorbance of the sample tube; *V*_T_ is the total volume of the sample solution (mL); *V*_S_ is the amount of the sample during the measurement (mL); and *m* is the fresh weight of the sample.

### Determination of malondialdehyde (MDA) content

The method for the determination of MDA content was referred to Lei et al. [31]. The method of configuration and grouping of the cucumber hydroponic solution was the same as that of the POD test. Three real leaves of cucumber plant at three-leaf-stage under salt stress were cut into strips and evenly mixed together to obtain 0.5 g broken leaves. To, it was added 5% trichloroacetic acid (5 mL), the homogenate was ground in the mortar, and transferred to a 10 mL centrifuge tube and centrifuged at 3000 rpm for 10 min. The volume of supernatant (*V*) of the sample extract was measured, out of which 2 mL (*V*_1_) was taken in a test tube and 0.67% thiobarbituric acid (TBA) prepared with 10% TCA was added. After mixing, the mixture was boiled in a water bath at 100 °C for 30 min and then centrifuged again after cooling. The supernatant was the test solution (*V*_2_) and its absorbance was determined by ultraviolet spectroscopy at 450 nm, 532 nm, and 600 nm.

The MDA concentration in the test solution was calculated by formula (7), and then the MDA content in the plant tissue was calculated according to formula (8);

7 $${\text{MDA concentration C }}\left( {\mu {\text{mol}}/{\text{L}}} \right) \, = { 6}.{45}\left( {A_{{{532}}} - A_{{{6}00}} } \right) - 0.{56}A_{{{45}0}} ,$$

8 $${\text{MDA content of the sample}}\left( {{{\mu mol} \mathord{\left/ {\vphantom {{\mu mol} g}} \right. \kern-0pt} g}} \right) = \frac{{C^{*} V_{2}^{*} V}}{{m^{*} V_{1}^{*} 1000}},$$

where, *A*_450_, *A*_532_, and *A*_600_ represent the absorbances at wavelengths of 450 nm, 532 nm, and 600 nm, respectively. *C* is the concentration of MDA in the liquid to be tested, *V* is the total volume of the extract (mL), *V*_1_ is the volume of the sample extract added in the liquid to be tested (mL); and *V*_2_ is the total volume of the liquid to be tested, which is 4 mL.

### Root absorption and translocation analysis

**Fig. 3:**
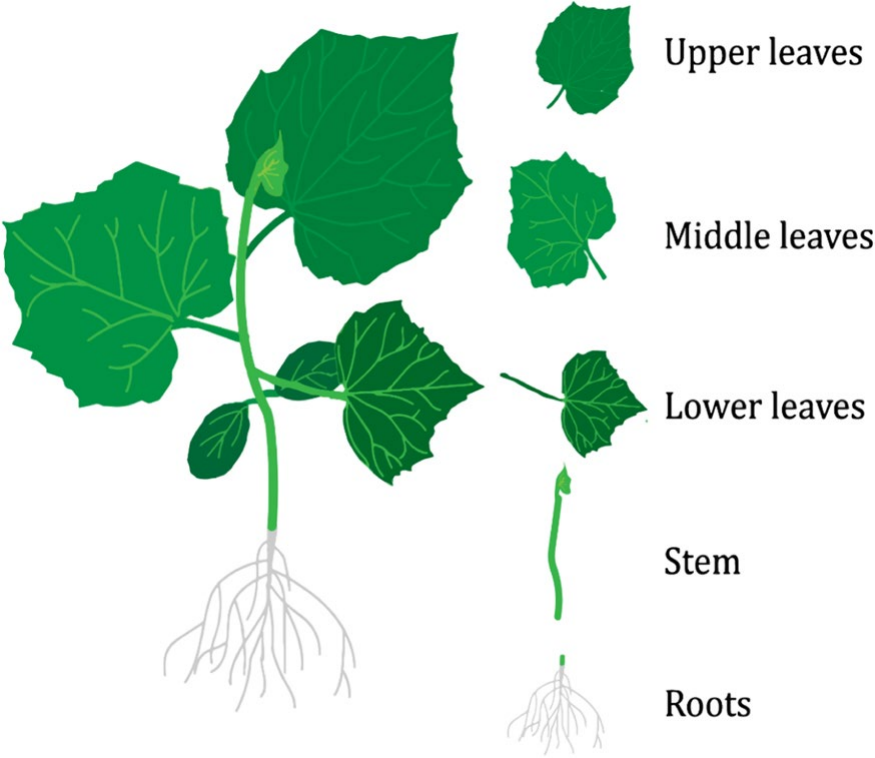
Structures of the cut parts of the cucumber plant

Cucumber seedlings with three true leaves in the culture medium were used as model plants for root absorption studies. The structural decomposition of cucumber is shown in Fig. 3. After the cucumber seedlings were removed from the culture medium, the roots were rinsed with deionized water, EB, EB@AM-zein, and EB@AM-zein-SA (having a concentration of 200 mg/L EB per 100 mL solution) and were packed into respective beakers and placed in an artificial climate chamber, with same planting conditions for 16 h/8 h of light/darkness, 28 ℃, and 80% humidity. After 24/48 h the cucumber seedlings were taken out, the roots washed with deionized water and dried. The roots, stems, lower leaves, middle leaves, upper leaves were cut and the quality of the five parts of cucumber seedlings were analyzed. They were ground in a centrifuge tube under liquid nitrogen and then 2 mL of 90% acetonitrile was added to the centrifuge tube containing the cucumber tissue. It was then centrifuged for 10 min at 3000 rpm and the supernatant was injected into a sample bottle through a 220 nm filter after withdrawing 1 mL supernatant from a disposable syringe for 10 min. The EB concentration in supernatant was determined by HPLC. The HPLC method applied here was similar to that of the photodegradation study.

### Insecticidal activity test

The leaves of Brassica Chinensis Shanghaiensis with dimensions of 2.5 × 2.5 cm were soaked in different concentrations of EB, EB@AM-zein, and EB@AM-zein-SA solutions for 1 min and then air-dried.

The 4th instar diamondback moth (*Plutella xylostella L.*) was placed in a culture dish having diameter of 6 cm. 10 pieces of *Plutella xylostella L.* were added into the culture dish on a filter paper. The rest of the procedure was the same as the above-mentioned methods. The experiments were repeated thrice for each group to eliminate errors and interference of other factors. The mortality rate of *Plutella xylostella* after 24 h was calculated by Abbott formula and *LC*_50_.

### Cytotoxicity test

The cytotoxicity of samples were evaluated on NIH3T3 cells by CCK8 assay. Select NIH3T3 cells in good condition, obtain a uniform cell suspension by routine passaging in a clean bench after sterilization, adjust the cell density to 104 cells/mL, and inoculate 100 μL per well into a 96-well plate, at 37 ℃, 5% CO_2_ incubator for 24 h. After 24 h, the medium was discarded, and 5 different concentrations (15.625, 31.25, 62.5, 125, 250 mg/L) of samples were added for detection. 3 replicates in each group, and design a blank group (only add fresh culture medium); after 24 h, discard the liquid in the well plate, add fresh culture medium to the corresponding well, and then add 5 μL of CCK8 solution to each well, incubate for 0.5 h in the dark in the incubator, measure the absorbance (OD value) of the corresponding well at 429 nm, and calculate the cell viability. The CCK8 value of cells in the blank group was considered to be 100% cell viability.

## Results and discussion

### Structure analysis

**Fig. 4:**
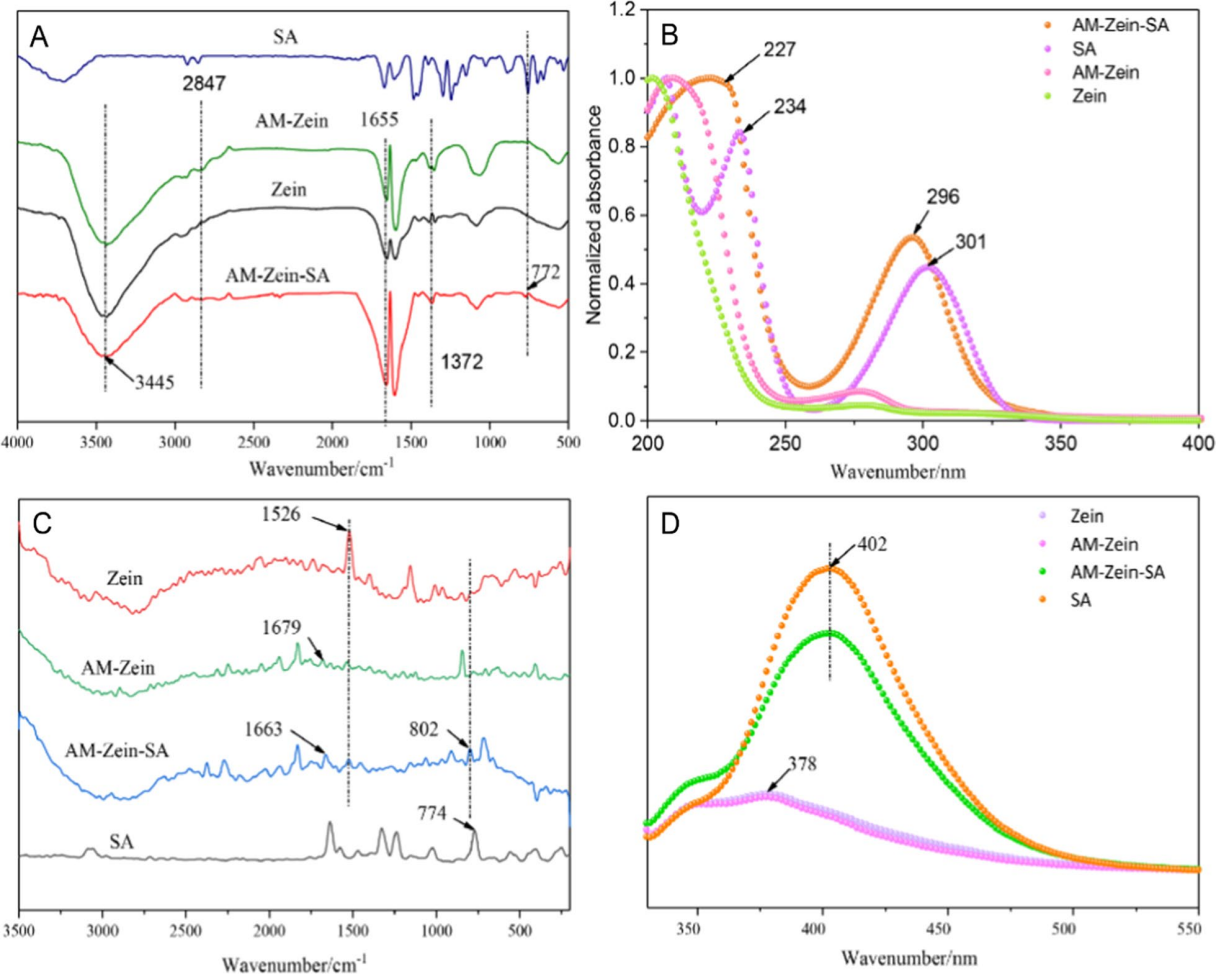
**A** FTIR, **B** UV, **C** Raman, and **D** Fluorescence spectra of zein, AM-zein, SA, and AM-zein-SA

The spectra of AM-zein and AM-zein-SA in Fig. 4A showed tensile and deformation vibrations of methylene groups of PEI-EDA at 1372 cm^−1^ and 2847 cm^−1^, which indicated the successful grafting of PEI-EDA onto zein-EDGE [32, 33]. The peak in the spectrum of AM-zein-SA at 3445 cm^−1^ was attributed to free primary amine group and of its intensity in AM-zein-SA was obviously decreased. The FTIR spectra of AM-zein and AM-zein-SA showed that the intensity of amide I peak in AM-zein-SA at 1655 cm^−1^ was higher than that of AM-zein, which could be ascribed to the reaction between amino group in AM-zein and the carboxyl group of SA. The successful grafting was indicated by the increased number of amide groups. The absorption peak for *ortho*-disubstituted benzene ring at 772 cm^−1^ in AM-zein-SA sample indicated the successful grafting of SA onto AM-zein-SA [34].

Figure 4B shows the UV spectrum of zein, AM-zein, AM-zein-SA, and SA. The UV absorption peaks of SA at 301 nm and 234 nm were attributed to the benzene ring and phenolic hydroxyl group [35]. Meanwhile, the absorption peaks for benzene ring in AM-zein-SA were slightly blue shifted to 296 nm, which could be attributed to the π-π stacking of the benzene ring of zein and the benzene ring of SA. Further, due to the changes in steric hindrance caused by the successful grafting of SA onto zein, the UV absorption peak of benzene ring of SA in AM-zein-SA was blue shifted [36]. The blue-shifting of the phenolic absorption peak at 227 nm confirmed the successful grafting of SA onto AM-zein.

In Fig. 4C, the bands at 1663 cm^−1^ and 1526 cm^−1^ in the AM-zein-SA Raman spectra could be attributed to the deformation of the amide I band (C=O stretching) and the amide II band (N–H) [37]. In addition, the band at 1679 cm^−1^ in AM-zein showed a *β*-folded structure, whereas a band at 1663 cm^−1^ in AM-zein-SA indicated a random coil conformation, which could be attributed to the hydrogen bonding between the phenolic hydroxyl group of salicylic acid and the protein. The result was destruction of the *β*-folded structure that caused random protein folding [38]. The band at 774 cm^−1^ in salicylic acid corresponded to the *ortho*-disubstituted benzene ring [39]. Salicylic acid was grafted on AM-zein due to the large number of amino groups in AM-zein that formed amide bonds and the π–π stacking between benzene–benzene rings. As a result, the absorption peak of *o*-disubstituted benzene ring was shifted to 802 cm^−1^ [40].

Figure 4D shows the fluorescence spectra of zein, AM-zein, AM-zein-SA, and SA. With the excitation wavelength set to 310 nm, the fluorescence spectra of SA, zein, AM-zein, and AM-zein-SA were recorded. The maximum emission wavelength of zein and AM-zein at 378 nm was due to the formation of a large number of l-phenylalanine and complex amino acid residues in zein [36]. The maximum emission wavelength of AM-zein-SA (at 310 nm excitation wavelength) was 402 nm, which was the same as the maximum emission wavelength of SA. These results showed that salicylic acid was successfully grafted onto AM-zein.

**Fig. 5:**
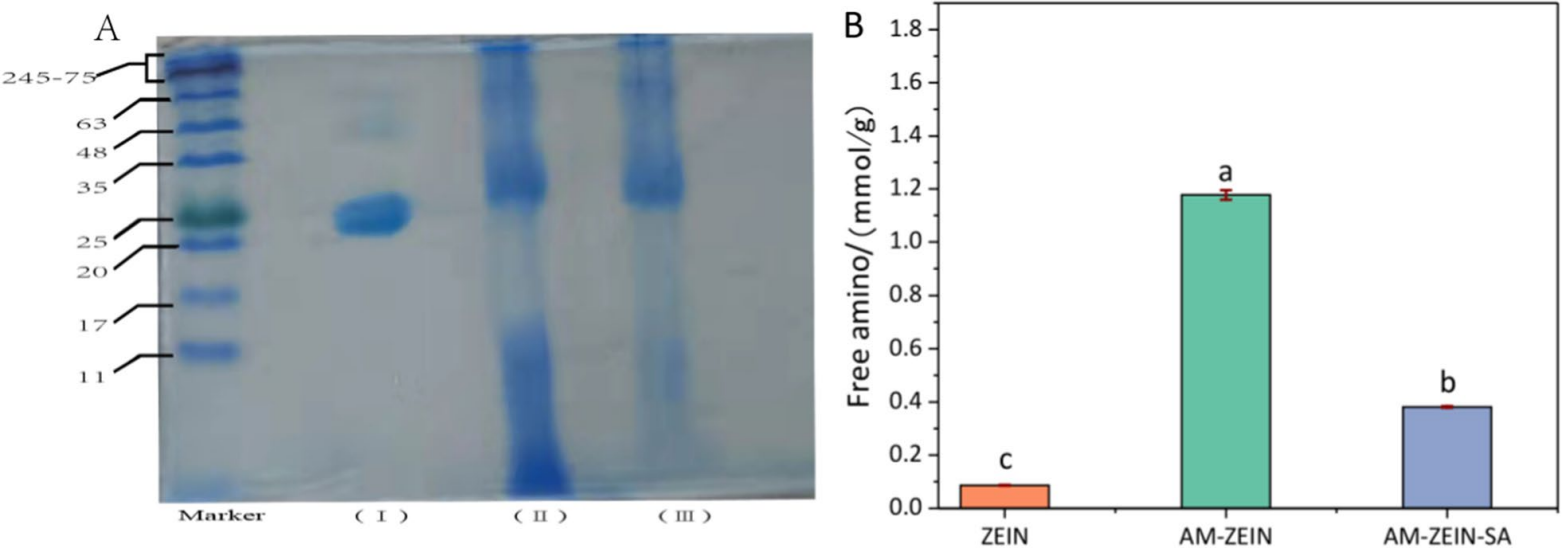
**A** SDS–PAGE gel electrophoresis results of the Marker, zein, AM-zein, and AM-zein-SA. **B** Free amino contents of zein, AM-zein, and AM-zein-SA. The experimental data were measured three replicates; a statistically significant difference between treatments compared with control is indicated by different small alphabets (a–c). Error bars indicate the least considerable value (LSD) at p ≤ 0.05 among the treatments

The results of SDS-PAGE electrophoresis of zein, AM-zein, and AM-zein-SA are shown in Fig. 5A. The molecular weight of zein was mainly concentrated in the range of 20–25 K and its concentration was 75%-85%, whereas the other bands that appeared at 48 K were of *β*-zein and *γ*-zein [41]. Grafting of PEI-EDA onto zein increased the range of molecular weight distribution of AM-zein. The molecular weight range shifted from 20–25 to 35–245 K, indicating the successful grafting of PEI-EDA on zein. The protein with molecular weight distribution below 17 K was hydrolyzed in AM-zein under alkaline conditions. AM-zein produced a large amount of hydrolyzed AM-zein with molecular weight below 11 K, due to protein chain scissions. The results of electrophoresis of AM-zein-SA showed that compared with AM-zein, there was a significant reduction in the distribution of AM-zein-SA below 17 K molecular weight. This occurred because AM-zein-SA was hydrolyzed to proteins having molecular weights less than 5 K under alkaline conditions, which were separated by dialysis. The molecular weight of AM-zein-SA did not increase appreciably as compared to that of AM-zein, since the molecular weight of SA was very less. Hence, there was no obvious displacement in SDS-PAGE electrophoresis after grafting.

The free amino contents of zein, AM-zein, and AM-zein-SA are presented in Fig. 5B. The free amino content of AM-zein grafted with PEI-EDA increased 13.6 times, as compared to the zein samples. At the same time, changes in the isoelectric point of zein caused by the grafting of a large number of amino groups solved the problem of aggregation of zein nanoparticles at the isoelectric point of around pH 6.2–6.8 and increased the AM-zein drug loading under near neutral conditions. On the other hand, the free amino content of AM-zein-SA was reduced by 67.6%, as compared with that of AM-zein. This indicated that the free amino groups in AM-zein reacted with the carboxyl groups of salicylic acid forming amide bonds, and some proportion of the free amino groups was reduced. The grafting ratio of SA was calculated according to formula [42] (9):

9 $${{DG} \mathord{\left/ {\vphantom {{DG} \% }} \right. \kern-0pt} \% } = {{\left( {C_{0} - C_{t} } \right)} \mathord{\left/ {\vphantom {{\left( {C_{0} - C_{t} } \right)} {C_{0} }}} \right. \kern-0pt} {C_{0} }}$$

where, DG represents the degree of grafting, *C*_0_ represents the free amino content in unreacted samples, and *C*_t_ represents free amino content of samples after reaction. Based on the number of free amino groups in AM-zein and AM-zein-SA, the grafting ratio of salicylic acid to AM-zein-SA was determined as 76.7%.

**Fig. 6:**
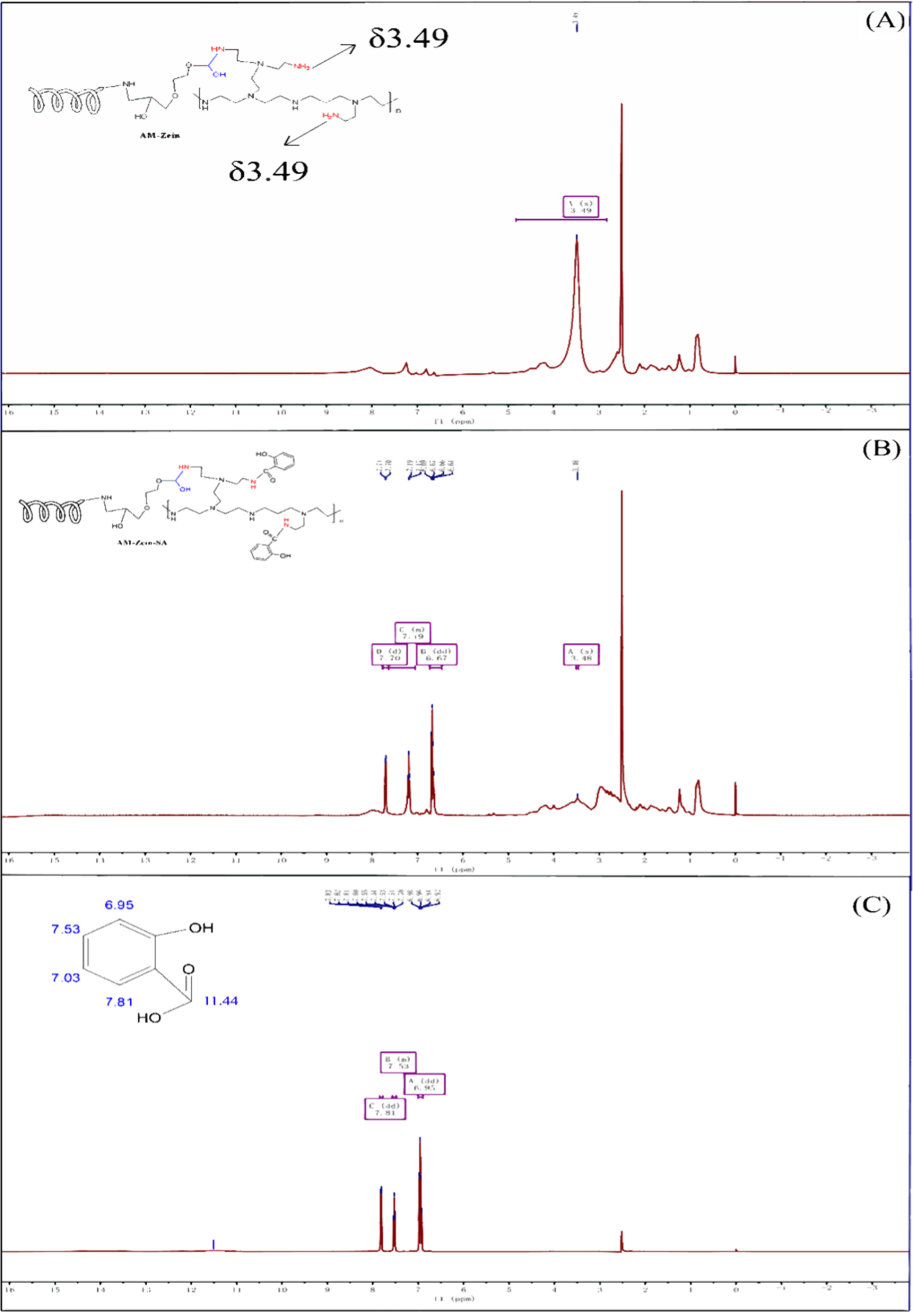
Proton Nuclear Magnetic Resonance Spectra of **A** AM-zein, **B** AM-zein-SA, and **C** SA

To further confirm the successful grafting of SA onto AM-zein, the structures of AM-zein, AM-zein-SA, and SA were further characterized by ^1^H NMR spectroscopy. Figure 6 shows the ^1^H NMR spectra of AM-zein, AM-zein-SA, and SA in deuterated-DMSO solvent, where the sharp proton signal at δ = 2.43 was due to the proton peak of deuterated-DMSO, peak at *δ* = 3.49 in AM-zein was attributed to the proton of free primary amine [43], and peaks between δ = 0−3.2 corresponded to the proton signals of 19 aliphatic amino acids that make up the zein molecule [44].

It can be seen from Fig. 6C that the chemical shifts of hydrogens of the aromatic ring of SA appeared at *δ* = 7.81, 7.53, and 6.94, whereas the peak at *δ* = 11.44 was due to the carboxyl group of salicylic acid. The intensity of the free amino peaks at *δ* = 3.49 in Fig. 6B decreased significantly. The carboxyl peak of SA at *δ* = 11.44 disappeared in the spectrum of AM-zein-SA due to the amidification reaction between AM-zein and SA. In addition, the benzene ring hydrogen of SA appeared at *δ* = 7.70, 7.19, and 6.67, which also proved the successful grafting of SA.

## SEM analysis

**Fig. 7:**
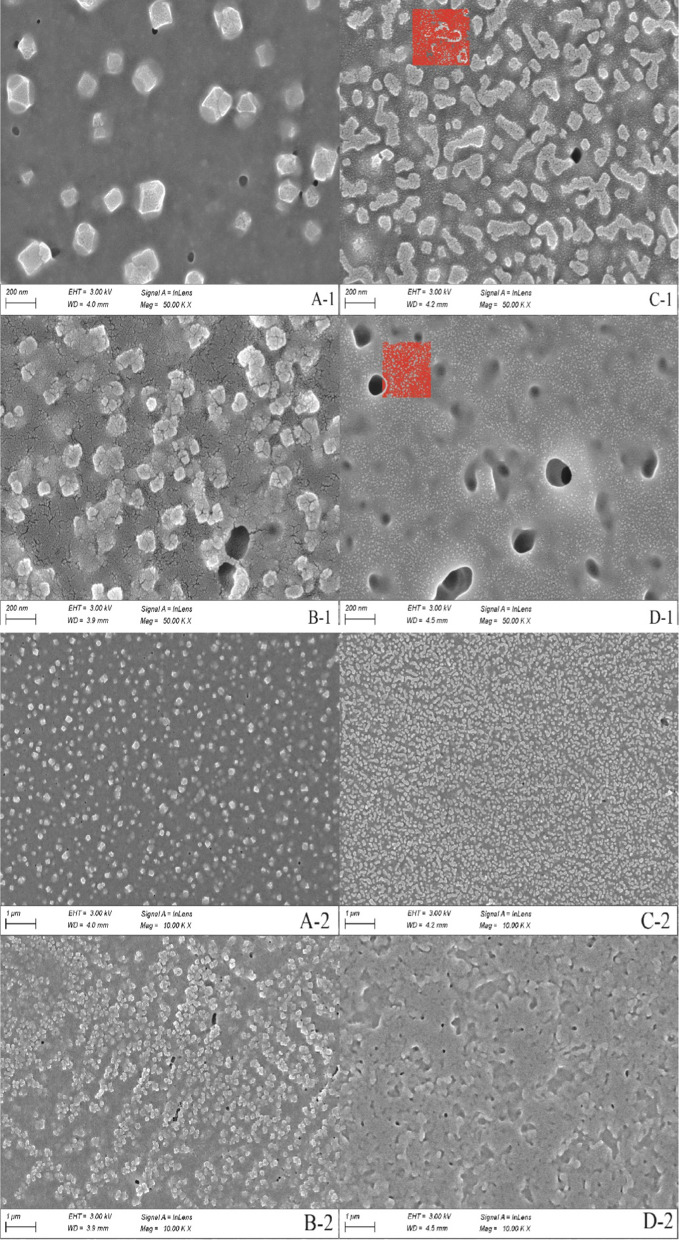
SEM images of **A** AM-zein, **B** AM-zein-SA, **C** EB@AM-zein, and **D** EB@AM-zein-SA

SEM analysis helped to determine the morphologies of the samples AM-zein, EB@AM-zein, AM-zein-SA, EB@AM-zein-SA. It can be seen from Fig. 7(A-1) that AM-zein had a smooth surface and irregular polyhedral shape. However, in Fig. 7(C-1), EB@AM-zein showed a change from a smooth surface to a brain-like folded and curled structure after loading of EB. Figure 7(B-1) showed that AM-zein-SA had a rough surface and irregular spherical shape. The adhesion between EB@AM-zein-SA particles could be seen in Fig. 7(D-1), which was due to the film-forming properties of zein [45]. EB@AM-zein-SA nanoparticles aggregated to form films, due to hydrogen bonding and hydrophobic interactions [46]. The red boxes in Figs. 7(C-1) and (D-1) showed the appearance of a nanoparticle with particle size significantly smaller than those of EB@AM-zein and EB@AM-zein-SA in EB@AM-zein and EB@AM-zein-SA samples. However, such smaller nanoparticles were not found in Fig. 7(A-1) and (B-1), and hence these nanoparticles with a particle size of about 3–5 nm were considered to be those of EB that were not encapsulated by the carrier.

**Fig. 8:**
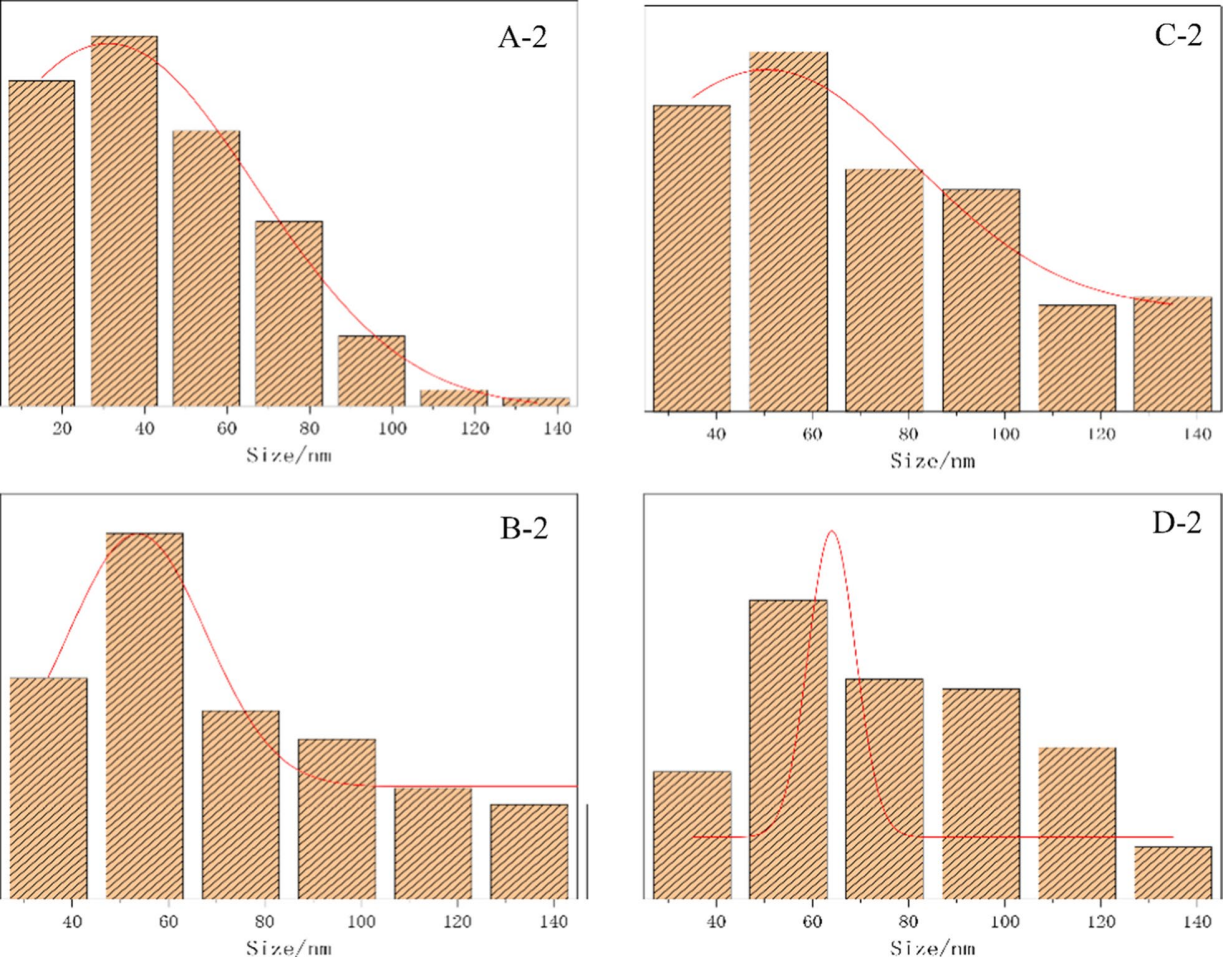
Particle size distribution of **A-2** AM-zein, **B-2** AM-zein-SA, **C-2** EB@AM-zein, and **D-2** EB@AM-zein-SA

Figure 8 is a statistical graph of particle size distribution of the samples from SEM images in Fig. 7(A-2, B-2, C-2, and D-2) by Image-Pro plus 6.0 software. The graph showed that the particle size of AM-zein-SA changed from 53 to 64 nm after loading of EB and this increase in particle size indicated that EB was successfully loaded onto the EB@AM-zein-SA carrier.

## Storage stability analysis

### pH stability analysis

Due to high surface energy, small size, and other physical properties, nano-particles tend to agglomerate and lose their nano-characteristics. Therefore, it is necessary to evaluate the storage stability of nano-pesticides under various conditions.

Zein is made up of more than 50% of non-polar amino acids (such as leucine, proline, and alanine) [47]. zein has hydrophobicity and the specific isoelectric point relative to those of amphoteric electrolytes. The isoelectric point of zein is usually between 6.2 and 6.8 [48]. The stabilities of the two nano-pesticides, EB@AM-zein and EB@AM-zein-SA, were evaluated at different pH values. Table 1 shows the data of particle sizes, potentials, and polydispersities of EB@AM-zein and EB@AM-zein-SA nanopesticides prepared by the simple anti-solvent method at different pH values. Both kinds of nano-pesticides had the smallest particle size at pH 7 and the zeta potential was more than 30 mV. This implied that the electrostatic repulsions between the nano-particles were strong and the nano-particles were stable and did not easily settle.

**Table 1:** Sizes and zeta potentials of EB@AM-zein and EB@AM-zein-SA nanoparticles

Sample	pH	Size/nm	Zeta potential/mV	PDI
EB@AM-zein	3	117.28 ± 11.86^a^	14.10 ± 2.53^d^	0.200 ± 0.011^d^
	5	76.41 ± 2.79^b^	48.60 ± 4.65^a^	0.228 ± 0.010^c^
	7	67.44 ± 2.60^c^	39.35 ± 1.06^b^	0.259 ± 0.007^b^
	9	77.18 ± 1.49^b^	23.80 ± 4.73^c^	0.299 ± 0.017^a^
EB@AM-zein-SA	3	118.20 ± 4.30^a^	30.34 ± 5.41^b^	0.268 ± 0.022^b^
	5	78.85 ± 3.05^b^	25.61 ± 3.18^c^	0.290 ± 0.004^a^
	7	73.98 ± 0.30^c^	40.81 ± 1.17^a^	0.217 ± 0.010^d^
	9	108.35 ± 1.80^b^	17.99 ± 3.19^d^	0.232 ± 0.021^c^

The experimental data were measured three replicates; a statistically significant difference between treatments compared with control is indicated by different small alphabets (a–d)

Error bars indicate the least considerable value (LSD) at p ≤ 0.05 among the treatments

The particle sizes of EB@AM-zein and EB@AM-zein-SA are mainly controlled by the potential [9]. The adsorption of EB on AM-zein and AM-zein-SA and the aggregation of nanoparticles with decrease in electrostatic force, affect the particle size of loaded nanoparticles. At pH 3, the increase in particle size is due to the aggregation of nanoparticles with lower potential. The decrease in potential leads to a decrease in adsorption of EB on the surface of AM-zein owing to electrostatic forces. Hence, the size of EB@AM-zein decreased at first and then increased as the pH changed from 5 to 9. At pH = 7, the equilibrium between the size of EB@AM-zein-SA nanoparticles and the drug loading rate was attained. The polydispersity indexes (PDI) of EB@AM-zein and EB@AM-zein-SA nanoparticles at all pH values were in the range of 0.2 to 0.3, indicating a high degree of uniformity and a small range of distribution of nanoparticles.

### Storage stability analysis

**Fig. 9:**
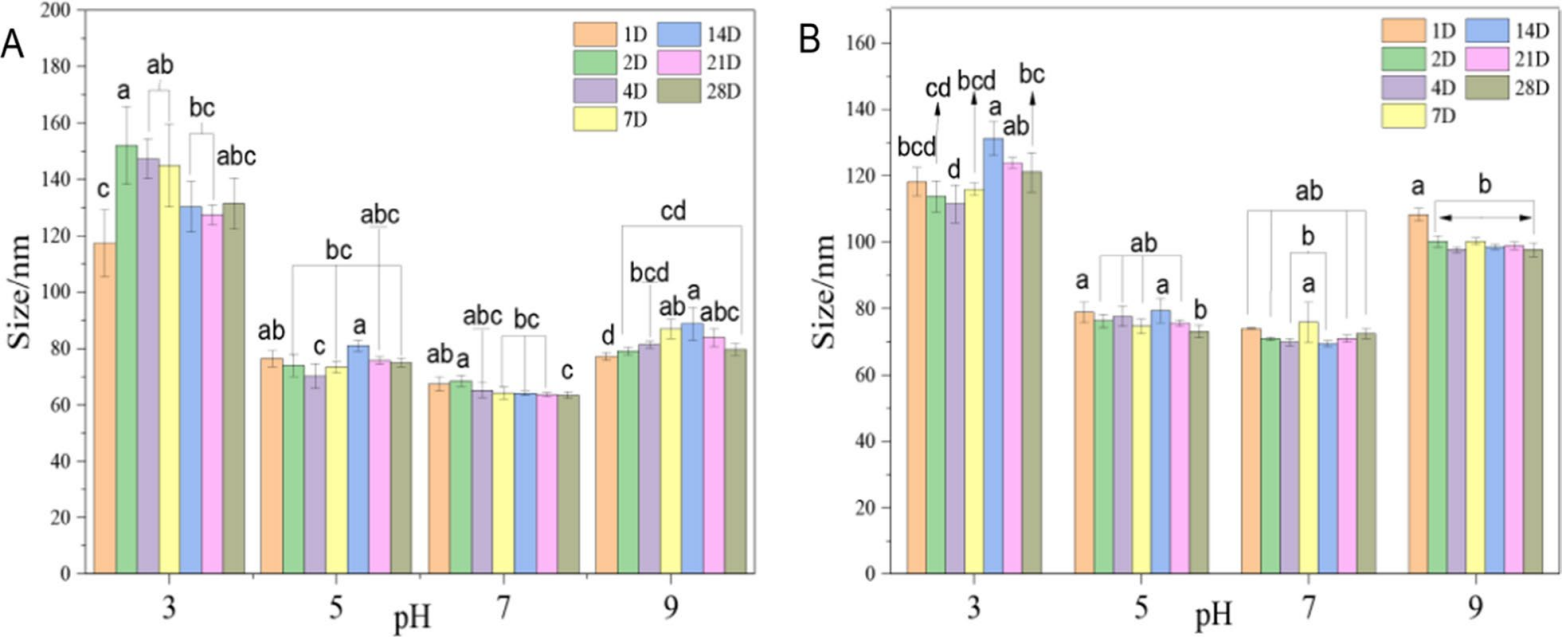
The particle sizes of **A** EB@AM-zein and **B** EB@AM-zein-SA during storage. The experimental data were measured three replicates; a statistically significant difference between treatments compared with control is indicated by different small alphabets (a–d). Error bars indicate the least considerable value (LSD) at p ≤ 0.05 among the treatments

The storage stability of a nano-pesticide preparation has great influence on its agricultural applications. It can be seen from Fig. 9 that the changes in particle sizes of the two kinds of nano-pesticides over a period of 28 days at room temperature were measured by laser particle size analyzer. It was found that the storage stability of EB@AM-zein-SA was good at pH 5, 7, and 9.

At pH 3, the particle size of EB@AM-zein-SA increased initially and then decreased with time. The reason was that the absolute value of zeta potential was low and the electrostatic repulsions between the particles were weak, which caused some AM-zein particles with weak electrostatic repulsions to settle down. A few number of unstable particles precipitated over time and the remainder were nanoparticles that were stably dispersed in water. In addition, at pH = 3, the strong acidity led to protein hydrolysis and consequently potential changes in the nano-system, allowing the high surface energy nanoparticles to aggregate. The particle size of EB@AM-zein-SA changed greatly, which was also due to the same reason.

## Leaf surface retention of EB preparation analysis

**Fig. 10:**
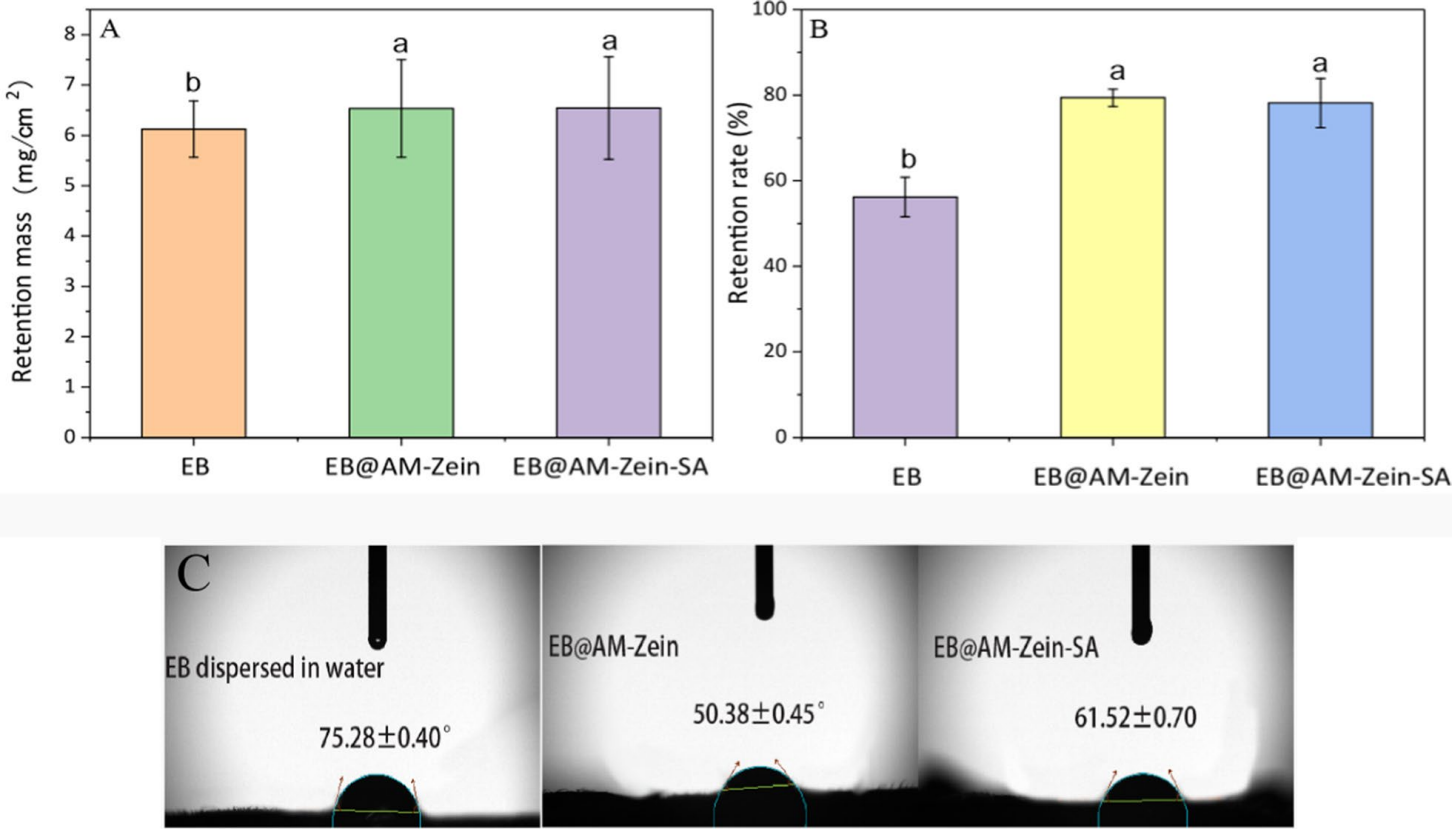
Interactions between the sample and leaf surface. **A** Retention mass of EB aqueous dispersion in water, EB@AM-zein, and EB@AM-zein-SA on cucumber leaves, **B** Simulation of the retention rates of EB aqueous dispersion, EB@AM-zein, and EB@ AM-zein-SA on cucumber leaves were after erosion by rain. **C** Contact angles of EB aqueous dispersion, EB@AM-zein, and EB@AM-zein-SA on cucumber leaves. The experimental data were measured three replicates; a statistically significant difference between treatments compared with control is indicated by different small alphabets (a, b). Error bars indicate the least considerable value (LSD) at p ≤ 0.05 among the treatments

Presently, the main way of applying pesticides is by spraying them on crop leaves. Hence, the retention rate of pesticide droplets on leaves can estimate the target efficiency of pesticides on leaves and the utilization rate of pesticides. Hence, experiments were designed to determine the interactions between pesticide droplets and the leaf surfaces and also to evaluate the targeting efficiency of pesticides, based on the retention of pesticide droplets on the leaf surface. Figure 10A shows the retention mass of EB aqueous dispersion, EB@AM-zein, and EB@AM-zein-SA on cucumber leaves. It can be seen from Fig. 10A that the retention rates of EB@AM-zein and EB@AM-zein-SA on leaf surfaces were 6.52% and 6.67% higher than those of EB. The retention of EB@AM-zein and EB@AM-zein-SA on the leaf surface was increased mainly due to the electrostatic interactions between the leaf surface and the positive charges. The polymer chain segment helped to prevent the breaking of the droplet [49] and the water film formed on the leaf surface was not easy to break and slide off.

Figure 10B was a simulation of the residual EB on cucumber leaves after rain washing. The retention rate of EB on cucumber leaves after washing was reduced by 43.83%. In comparison to that, EB@AM-zein-SA could effectively improve the anti-scouring ability of EB by 39.09%. Compared with EB, EB@AM-zein-SA had shown great improvement. The reason why EB@AM-zein-SA could improve the erosion resistance of EB was the film-forming property of zein. The films formed by EB@AM-zein and EB@AM-zein-SA have strong adhesive property after liquid drops dried. The bonding mechanism in zein-based adhesives was mainly due to hydrogen bonding and hydrophobic interactions as well as electrostatic forces. Both, AM-zein and AM-zein-SA contained a large number of free amino groups and phenolic hydroxyl groups in SA, which could form hydrogen bonds with cucumber leaves to improve their adhesivity [50]. In addition, a large number of positive charges on the carrier could electrostatically interact with the negative charges on cucumber leaf surfaces.

## Wettability of blade surface

The contact angle made by a droplet reflects the wettability at the tri-phasic interface. The main component of the cucumber leaf surface is a waxy layer with long carbon chain fatty acids [51]. They are relatively strongly hydrophobic, and a higher contact angle would indicate easy sliding off from the hydrophobic blade and consequently loss of the target pesticide. Presently, many dispersants and wetting agents are added to the pesticide preparation to increase the duration of deposition of pesticides on crop leaves. However, these auxiliaries cause serious pollution to the environment.

The contact angles of EB, EB@AM-zein, and EB@AM-zein-SA droplets on cucumber leaves (Fig. 10C) were 75.00°, 50.05°, and 61.03°, respectively. The contact angles of the two zein-based drug-loaded particles on cucumber leaf surface were smaller than that of EB aqueous dispersion, which indicated that the two kinds of amino-modified zein carriers obviously improved the wettability of EB on cucumber leaf surface. As compared with AM-zein, the contact angle of AM-zein-SA was larger, since SA is hydrophobic. At the same time, the free amino groups on AM-zein were involved in the grafting of AM-zein with SA. The decrease in the number of free amino groups reduced the hydrogen bonding interactions between AM-zein-SA and leaves as compared to that of AM-zein, and consequently decreased the wettability of AM-zein-SA on the leaves. In this study, nanoscale pesticide carriers, AM-zein and AM-zein-SA, were used to improve the dispersibility of EB in water and reduce the use of petrochemical solvents and dispersants, and had good spreadability on leaf surface.

## Resistance of cucumber to salt stress

**Fig. 11:**
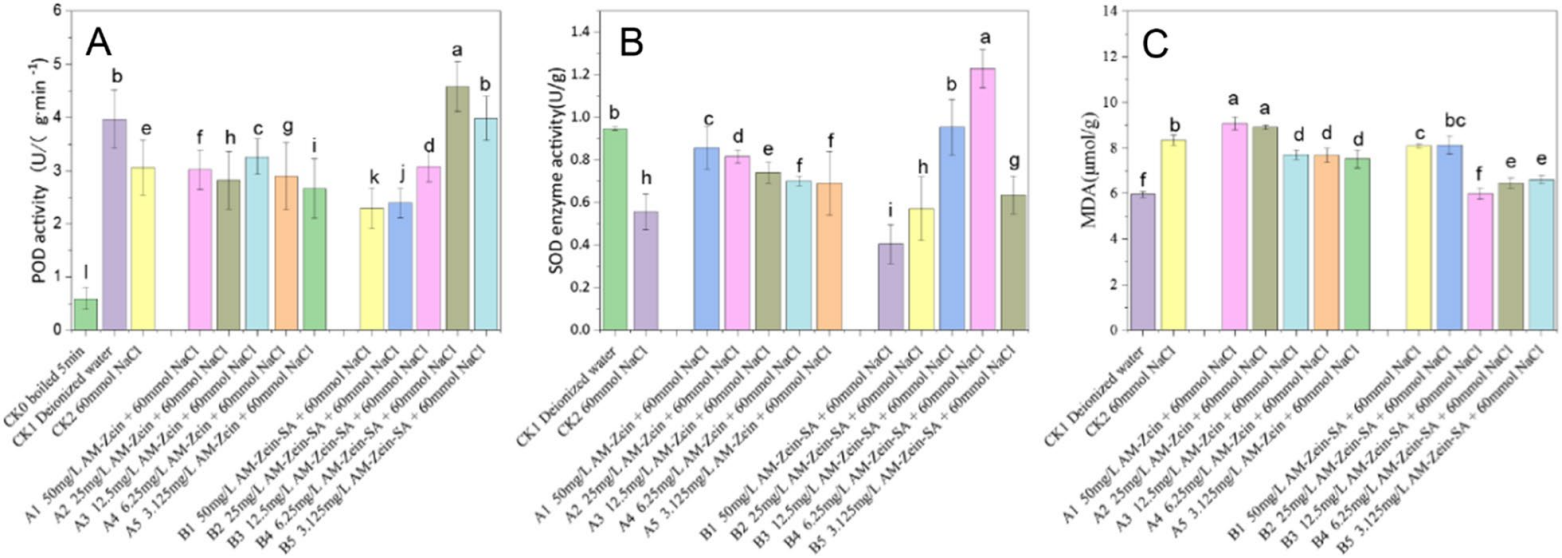
Changes in the contents of antioxidant enzymes, POD and SOD, in cucumber under 60 mmol salt stress of different concentrations of **A** AM-zein and **B** AM-zein-SA and **C** the MDA content depicts the degree of damage to the cell lipid membrane. The experimental data were measured three replicates; a statistically significant difference between treatments compared with control is indicated by different small alphabets (a–l). Error bars indicate the least considerable value (LSD) at p ≤ 0.05 among the treatments

Figure 11A shows the changes in POD activity of cucumber leaves under the stress of 60 mmol NaCl at different concentrations of AM-zein and AM-zein-SA after 1 d culturing in hydroponic medium. The POD activities of 1–5 in Group A were similar to that of CK_2_, which showed that the POD activity of cucumber under salt stress did not increase due to AM-zein. The decrease in POD activity resulted in a large number of ROS, which could not be timely decomposed [52]. A large number of ROS attacked the lipid membrane of cucumber cells, resulting in a large amount of material flow from the cells and the cucumber cell growth was adversely affected.

The POD activity of group 1–5 increased initially and then decreased in the samples treated with AM-zein-SA of different concentrations. When treated with 6.25 mg/L concentration of AM-zein-SA, the POD activity of the B4 group increased by 49.67%, compared with that of CK_2_ group. The POD activity in cucumber was higher than that in CK_1_ Group, whereas the POD activities in B1 and B2 groups were even lower than that in CK_2_ group. The reason was explained by Rajabi et al. [53], who treated sorghum with different concentrations of salicylic acid. The POD activity of sorghum under low to high concentrations of SA showed a trend of an initial increase followed by a decrease. On the other hand, a higher concentration of AM-zein-SA showed the same trend as that of higher SA concentration. Results showed that with decrease in the concentration of AM-zein-SA, the AM-zein-SA carrier could obviously decrease the POD activity caused by NaCl stress at an optimal concentration and increase the POD activity of cucumber under salt stress.

Figure 11B shows the SOD activity of cucumber under salt stress. Compared with CK_1_, the SOD activity of cucumber in CK_2_ group decreased obviously. The increase of SOD activity of No. 1–5 in Group A could be due to the stimulation of zein grafted PEI-EDA. The study of Abedini et al. [54] showed that plants respond to stress under exogenous stimulation and increase the antioxidant activities of different enzymes in response to environmental changes. Another possible reason could be that the amino acids that make up zein contain free proline residues on the surface of zein. As a substance for water retention and drought resistance in plants, proline decreased the dehydration of cucumber cells, and thus reduced the impact of salt damage on SOD enzyme activity [55]. With decrease in AM-zein concentration, SOD enzyme activity also decreased. This showed that the SOD enzyme activity had significant correlation with the concentration of AM-zein. The improvement in SOD enzyme activity helped to reduce the damage of ROS to plant cells under salt stress and reduce the adverse effects of salt stress on the growth of cucumber.

It can be seen from the samples treated with different concentrations of AM-zein-SA (No. 1–5 in group B) that the SOD enzyme activities of samples No. 1–5 showed a trend of first increase and then decreased. When treated with a concentration of 6.25 mg/L AM-zein-SA, the SOD enzyme activity of cucumber was higher than those of the CK_1_ and CK_2_ group by 29.96% and 121.22%, respectively. It was evident that the SOD activities of the B1 and B2 groups were even lower than those of the CK_2_ group. This indicated that the treatment with high concentrations of AM-zein-SA also inhibited the SOD enzyme. However, with decrease in AM-zein-SA concentration, the AM-zein-SA carrier could significantly decrease the decreasing rate of SOD enzyme activity caused by NaCl stress on cucumber. At optimal concentration, it could also increase the SOD activity of cucumber. Mahdi et al. [56] concluded the same when salicylic acid was to applied to corn (KSC400 var.) under drought stress. In other words, treatment with appropriate concentration of salicylic acid could significantly improve the activity of plant antioxidant enzymes. Further, with treatment of high concentration of SA, SOD enzyme activity was inhibited.

Figure 11C showed the MDA contents of cucumber tissues under salt stress. Compared with cucumber cultured in deionized water, the MDA content of cucumber under salt stress was increased by 36.6%. The cucumber had a higher MDA content than the cucumber under salt stress, which could be due to the fact that PEI-EDA in AM-zein caused oxidative stress in the cells. More ROS were generated that attacked the cell lipid membrane thus increasing the oxidative stress [57]. MDA decreased with decrease in AM-Zein concentration, with which the degree of oxidative stress response also decreased accordingly. Combination of the POD and SOD enzyme activities of AM-zein-SA at the same concentration showed that the MDA content was inversely proportional to the SOD and POD enzyme activities. ROS caused decrease in the MDA content, which was generated in the oxidation of cellular lipid membranes [58]. On treatment with 6.25 mg/L AM-zein-SA, the MDA content was comparable to the MDA content in deionized water. As compared with CK_2_ group, MDA content was reduced by 22.66%. This effectively reduced the oxidation of the cell lipid membrane by ROS, which resulted in the outflow of the contents from the lipid membrane of the cell, thus reducing the risk of plant damage.

## Seed Germination Analysis under Salt Stress

**Fig. 12:**
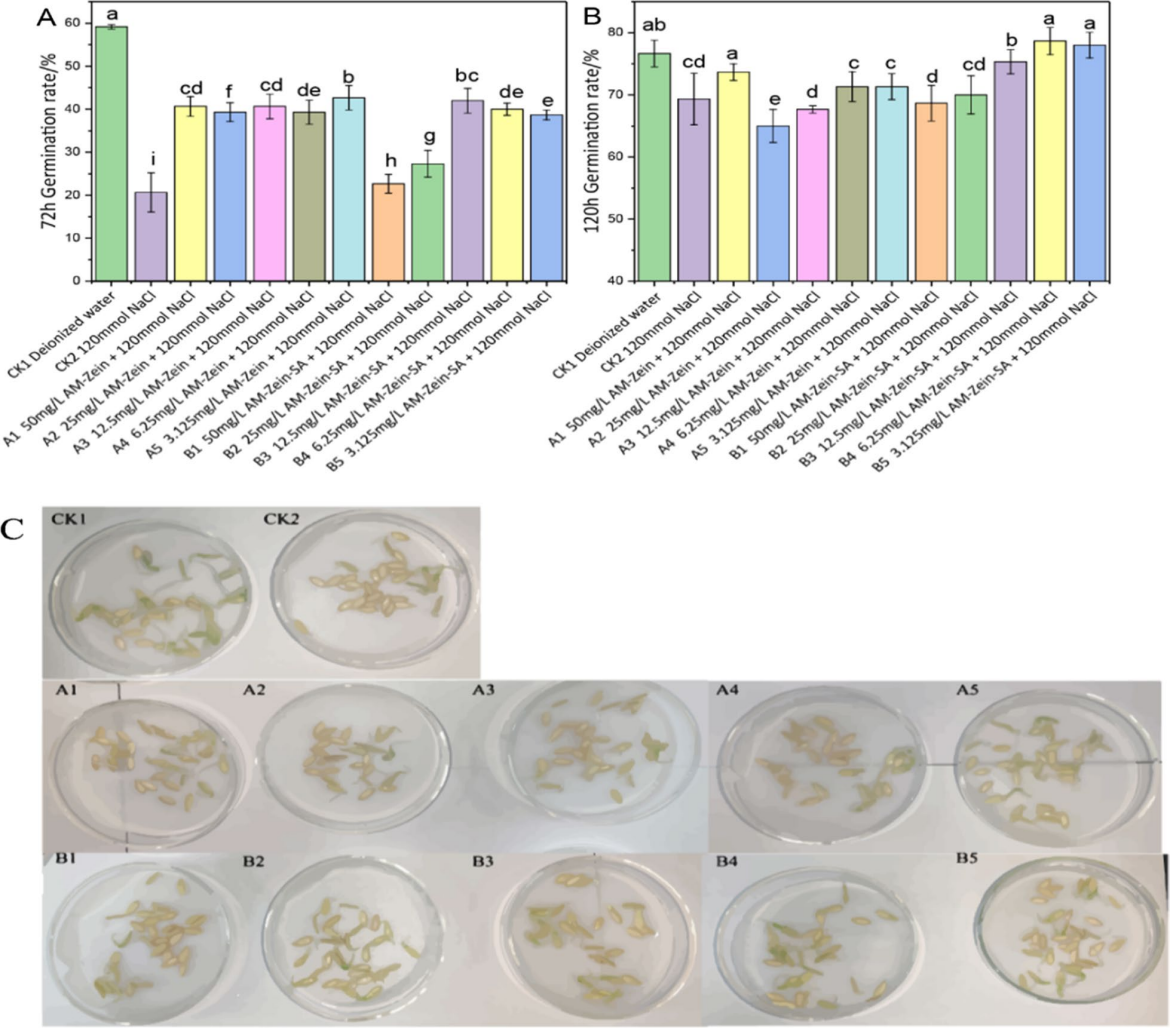
Germination rate of cucumber seeds under salt stress for **A** 72 h and **B** 120 h in different concentrations of AM-zein and AM-zein-SA solutions and **C** Germination rate and germination potential of cucumber seeds treated with different concentrations of carrier under 120 mmol salt stress after 72 h. The experimental data were measured three replicates; a statistically significant difference between treatments compared with control is indicated by different small alphabets (a–i). Error bars indicate the least considerable value (LSD) at p ≤ 0.05 among the treatments

Results of Fig. 12A and B showed that the germination rate increased from 59.17% to 76.67%, an increase by 17.5% in 72–120 h CK_1_ treatment group. The germination rate of CK_2_ group increased from 20% to 69.33%, an increase by 48.66%. It was evident that 120 mmol NaCl salt stress prolonged the germination time of cucumber. Moreover, the germination rate of cucumber seeds after treatment for 72 h increased from 20.67% in the CK_2_ group to 40% in the B4 Group, which corresponded to an increase of 93.51%. After treatment with 6.25 mg/L of AM-zein-SA under salt stress, the germination rate increased from 69.33% to 78.67% after 120 h of treatment. After treatment with 6.25 mg/L AM-zein-SA for 120 h, the germination rate of cucumber seeds was higher than that of the control group treated with deionized water. This indicated that 6.25 mg/L AM-zein-SA could effectively alleviate the damage caused by salt stress on cucumber seeds and also accelerated the germination of cucumber seeds. Liu et al. [59] studied the germination of rice seeds under salt stress. The experimental results showed that salt reduced the agricultural productivity by inhibiting seed germination. The inhibition was attributed to the accumulation of superoxide and MDA in seeds under salt stress. The activity of amylase was decreased, which eventually led to the inhibition of rice seed germination. Combined with the changes in SOD and POD activities with the use of 6.25 mg/L AM-zein-SA in this study, it was reasonable to speculate that AM-zein-SA alleviated the damage to cucumber seeds caused by salt stress. This was achieved by reducing the accumulation of superoxide and the damage to the cell lipid membrane, thus improving the germination of cucumber seeds.

By treatment of high concentration of AM-zein-SA for 72 h, the germination rate of cucumbers in the B1 and B2 groups were only 22.67% and 27.33%, which were significantly lower than those of the experimental group with the same concentration of AM-zein. The reason for decrease in germination rate was that the high concentration of AM-zein-SA in the treatment caused phytotoxicity and inhibited the germination and growth of cucumber seeds. The potential for germination under salt stress for 72 h is shown in Fig. 12C. An interesting phenomenon could be seen, wherein the germination potential of cucumbers under salt stress in the AM-zein group was stronger than that of AM-zein-SA. Combining this with the electrophoresis of AM-zein showed that AM-zein was in alkaline state. The lower part of zein was hydrolyzed into shorter peptides and the SOD enzyme activity of the group treated with AM-zein was higher than that of the CK_2_ group. AM-zein displayed nano-priming mechanism for cucumber under salt stress. Naveen et al. [60] found that when wheat seeds were primed with Fe_2_O_3_ nanoparticles, the germination rate of wheat seeds increased by 41.6% when Fe_2_O_3_ concentration was 200 mg/L. Aydin et al. [61] studied the self-protection mechanism of soybean under salt stress. Soybean synthesizes shikimic acid from aromatic amino acids such as phenylalanine, tyrosine, and tryptophan and finally synthesizes SA to regulate the physiological and biochemical reactions under stress. Zein contains large amounts of aromatic amino acids, which are hydrolyzed into short peptides under alkaline conditions. They are more conducive for the absorption of cucumber seeds under salt stress and improve the resistance of cucumber to salt stress. Moreover, it was evident that the SOD enzyme activity of cucumber when treated with AM-zein was improved. Scavenging of ROS by SOD enzyme helped to adapt to stressed environment and improve the germination rate.

## pH-responsive release rates of EB@AM-zein and EB@AM-zein-SA

**Fig. 13:**
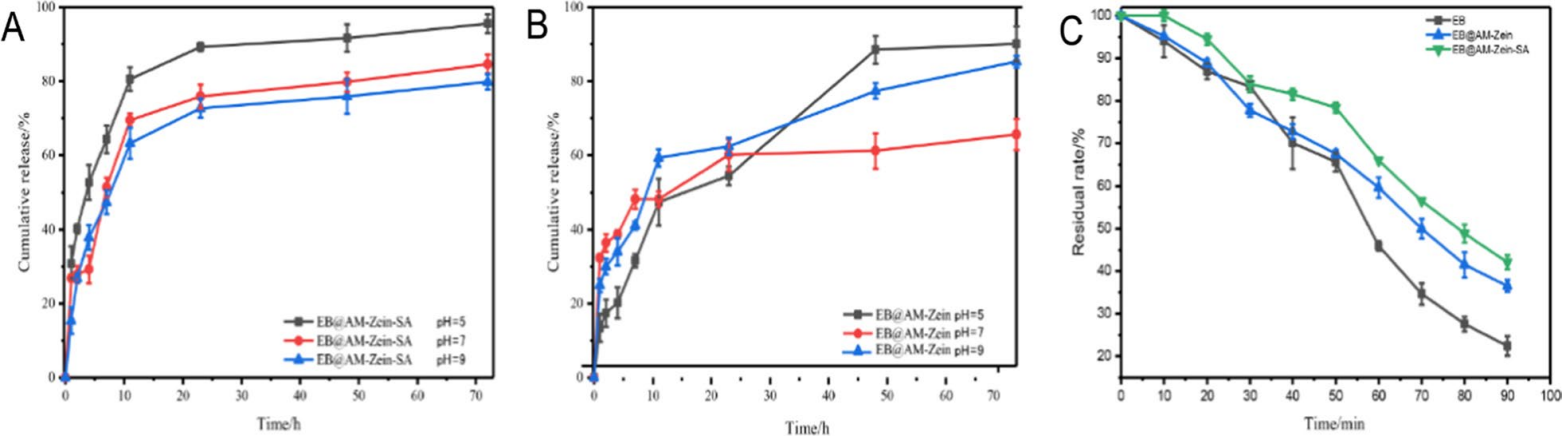
Slow release curve of **A** EB@AM-zein-SA and **B** EB@AM-zein at different pH values, **C** Photodegradation curve of EB in EB@AM-zein and EB@AM-zein-SA solutions

The in vitro release rates of drugs from two different carriers EB@AM-zein and EB@AM-zein-SA in 25% ethanol–water solution at pH values of 5, 7, and 9, were studied. It was evident from Fig. 13A and B that EB@AM-zein-SA showed obvious response release effect in acidic medium at pH 5 after 72 h of sustained release. The main possible reason could be the electrostatic forces between AM-zein-SA and EB. The free amino content on the surface of AM-zein-SA decreased after the reaction with SA, whereas the number of positive charges and force of binding with EB decreased after the protonation of the free amino on the surface of AM-zein-SA in acidic medium, which accelerated the release of EB in acidic conditions. The drug release kinetics of EB@AM-zein-SA was found to be consistent with the first-order model.

In the first-order fitting equation *y* was the percentage of drug dissolved during time *x*. According to the first-order kinetic model of drug release, the amount of EB released from AM-zein-SA was inversely proportional to the initial drug loading of the system within a certain period of time. The release rate of EB was proportional to the surface area of AM-zein-SA. The release rate of EB was inversely proportional with thickness of the capsule wall. The curve for cumulative release of EB as a function of time showed negative exponential growth [62]. The sustained-release process of EB@AM-zein could be obtained by fitting the kinetic equation of drug release, which was highly consistent with the Korsmeyer-Peppas model. The Korsmeyer-Peppas model was suitable for fitting of drug release data from microcapsule or microsphere formulations. According to the Korsmeyer-peppas model, when the C_1_ value of the spherical formulation was less than 0.43, the drug release followed Fick diffusion. Non-Fick diffusion mechanism was 0.43 < 0.857 [63, 64]. The coefficients C_1_ for both, EB@AM-zein and EB@AM-zein-SA release curve fitting Korsmeyer-peppas models, were less than 0.43 and it was evident that the release of EB was controlled by Fickian diffusion. Concentration was the main factor that influenced the EB release. However, at pH 5 and pH 9, it followed the Fickian diffusion in type space and at pH 7 it followed the Fickian diffusion in Euclidean space. Drug release at three different pH conditions was concentration-dependent. Table 2 shows fitting of drug release kinetics equation of EB@AM-zein-SA at different pH values.

**Table 2:** Fitting of drug release kinetics equation of EB@AM-zein-SA at different pH values

Sample	Fitting model	pH	Fitting equation	C_0_	C_1_	R^2^
EB@AM-zein	Zero-order	5	y = C_0_*x	1.199	–	0.8676
		7		0.9300	–	0.7363
		9		0.5669	–	0.5079
EB@AM-zein-SA		5		0.7581	–	0.6198
		7		0.9228	–	0.6092
		9		0.8842	–	0.6072
EB@AM-zein	First-order	5	y = C_0_(1-exp^-C1x^)	45.38	1810	0.2178
		7		75.79	0.1459	0.9000
		9		48.85	5.381E9	0.6607
EB@AM-zein-SA		5		90.34	0.2341	0.9310
		7		80.88	0.1584	0.9468
		9		76.25	0.1655	0.9869
EB@AM-zein	Korsmeyer-peppas	5	y = C_0_*x^C^1	13.30	0.4630	0.9740
		7		32.89	0.1665	0.9869
		9		25.18	0.2896	0.9824
EB@AM-zein-SA		5		42.29	0.2068	0.8787
		7		29.47	0.2638	0.9254
		9		26.41	0.2790	0.9243

## Anti-ultraviolet property

It can be seen from Fig. 13C that the protective effects of EB@AM-zein-SA on EB were 16.7% and 46.81% higher than those of EB@AM-zein and EB under UV lamp irradiation for 90 min. The main mechanisms of UV absorption by zein were divided into the following two types. 1. EB was physically blocked by zein base carrier. 2. Zein on exposure to ultraviolet light at high energy led to the breaking of chemical bond and a large number of non-polar amino acid residues were exposed. These non-polar amino acids had strong UV absorptivity due to their benzene ring structure [9, 65]. The phenolic hydroxyl group of salicylic acid in AM-zein-SA had strong UV light absorptivity. SA was oxidized to the yellow–brown colored carboxyl-anthraquinone after absorbing ultraviolet light, which reduced the sensitivity of EB to ultraviolet light. As compared with the aqueous dispersant of EB, EB@AM-zein-SA could obviously improve the anti-UV ability of EB@AM-zein-SA.

### Root absorption and translocation analysis

Improving the absorption and transportation of non-systemic insecticide is one of the major scientific problems that the researchers should study and solve. This can be achieved by direct modification of pesticide molecules to improve their absorption properties, such as grafting of glucose with pesticides to enhance the conduction of non-systemic pesticides inside the phloem [9, 66]. The aim of this study was to improve the uptake and transportation of the carrier AM-zein-SA by grafting plant endogenous hormone SA.

**Fig. 14:**
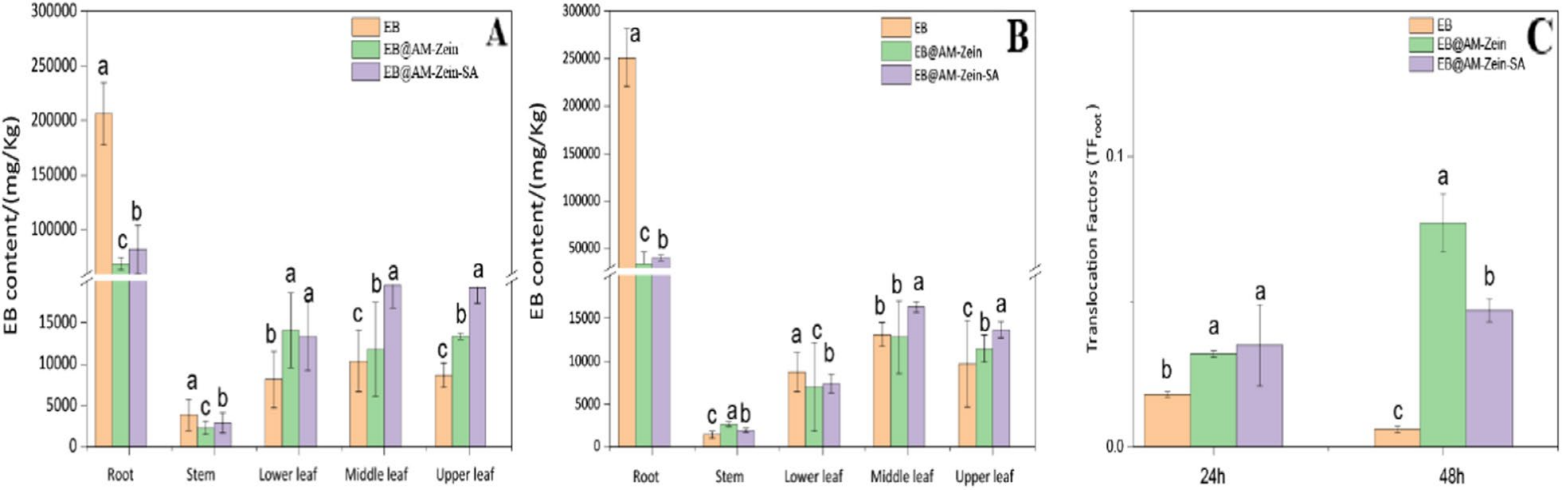
**A, B** Distribution of EB in cucumber after 24/48 h of application, **C** Translocation Factors (TFs) of EB/EB@AM-zein/EB@AM-zein-SA in cucumber with root treatments. The experimental data were measured three replicates; a statistically significant difference between treatments compared with control is indicated by different small alphabets (a–c). Error bars indicate the least considerable value (LSD) at p ≤ 0.05 among the treatments

EB is a non-systemic pesticide with low cytocompatibility and cannot be absorbed and transported by plants. However, it has strong permeability and some amount of EB can enter through the plant surface by osmosis. Figure 14A showed that the EB content in the EB treated group was much higher than those in the EB@AM-zein and EB@AM-zein-SA treated groups, which could be attributed to the higher octanol–water partition coefficient of EB as a result of its weak polarity. EB@AM-zein and EB@AM-zein-SA with nano-pesticides could increase the water solubility of EB, decrease the octanol–water partition coefficient of EB, and reduce the enrichment of EB in cucumber roots [67].

As seen from Fig. 14A, B, the highest percentage of EB in the 24 h treatment group was from the middle and upper leaves of cucumber plants. The EB content of EB@AM-zein-SA in the upper leaves was 88.53% and 122.65% higher than those in the group treated with EB and EB@AM-zein, respectively. As shown in Fig. 14C, translocation factor (*TF*) was also introduced to evaluate the capability of the cucumber to transfer organic chemicals. *TF*_root_ = *C*_stem_/*C*_root_ for treatment through roots [68]. The average *TF*_root_ of EB in EB@AM-zein-SA was almost 2 and 7.6 times those in EB after 24/48 h of treatment, respectively. This indicated higher capability of EB@AM-zein-SA to translocate EB from roots to stem. The distribution of the three EB preparations in cucumber at 24 h showed more accumulation of EB by EB@AM-zein-SA than the other three EB preparations in the stems and three leaves of cucumber plants.Compared with EB, EB@AM-zein-SA showed increase by 77.22% and 19.08%, respectively, in the stems of the groups treated for 24 h and 48 h. These results showed that EB@AM-zein-SA could promote the absorption and transportation of EB in cucumber. The possible reasons for the accelerated transportation of EB@AM-zein-SA in cucumber were as follows. (1) SA treatment could increase the accumulation and transportation of cadmium in the plant shoot, that is, SA treatment increased the accumulation of toxic substances and also led to the rapid accumulation of EB in cucumber [69]. (2) SA treatment increased stomatal conductance, efficiency of water use, and transpiration of cucumber. EB@AM-zein-SA could be transported to the aerial parts of cucumber more quickly [70].

### Insecticidal activity analysis

The insecticidal activities of EB, EB@AM-zein, and EB@AM-zein-SA against *Plutella xylostella* were evaluated by *LC*_50_ values and compared. In Table 3, the virulence regression equations of EB, EB@AM-zein, and EB@AM-zein-SA were obtained. The *LC*_50_ values were 0.67, 0.84, and 0.70, respectively.

**Table 3:** Insecticidal activities of different EB samples against *Plutella xylostella*

Sample	Toxicity equation	*LC*_*50*_/(mg/L)	95%	*R*^2^
EB	y = 1.7254x + 5.3047	0.67	0.48–0.93	0.990
EB@AM-zein	y = 1.8528x + 5.1429	0.84	0.63–1.11	0.967
EB@AM-zein-SA	y = 1.4758x + 5.2298	0.70	0.48–1.02	0.925

It can be seen that the insecticidal activity of EB@AM-zein-SA was not affected, as compared with EB. The increase of insecticidal activity of EB@AM-zein-SA compared with EB@AM-zein could be due to the decrease in acetylcholinase activity in P. xylostella caused by SA and the increase in insecticidal activity due to the subdued detoxification of EB [71].

### Cytotoxicity analysis

**Fig. 15:**
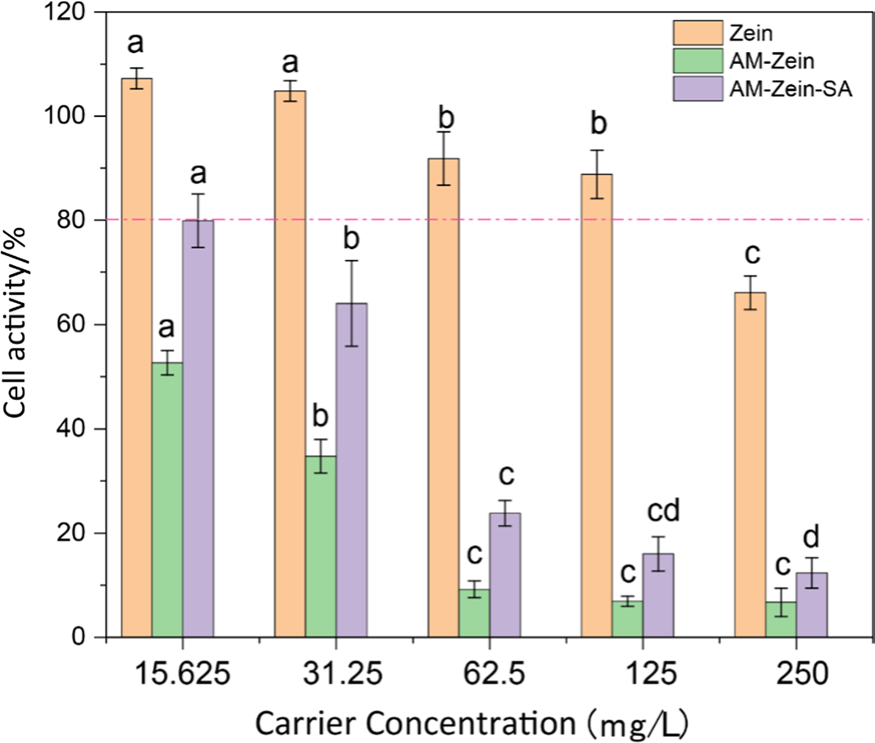
Cytotoxicity of samples zein, AM-zein, AM-zein-SA. The experimental data were measured three replicates; a statistically significant difference between treatments compared with control is indicated by different small alphabets (a–d). Error bars indicate the least considerable value (LSD) at p ≤ 0.05 among the treatments

Results of Fig. 15, cell activity was greater than 80% at a concentration of zein below 125 mg/L, indicating that the zein material itself had good biocompatibility and could be regarded as having no obvious cytotoxicity at this concentration. Combined with chapter 3.6 and 3.7, it can be concluded that AM-zein-SA has the best effect at the concentration of 6.25 mg/L. The cell activity of AM-zein-SA was 79.88% at the concentration of 15.625 mg/L, which indicated that AM-zein-SA had higher biosafety when used below this concentration. The cytotoxicity of AM-zein-SA was 34.05% lower than that of AM-zein. Some researchers think that the high cytotoxicity of PEI may be due to its ability to induce apoptosis. Further studies by Hall et al.[72] found that PEI promotes proton leakage from mitochondria and inhibits the electron transport chain in a concentration-and time-dependent manner, leading to apoptosis. M K et al. [73] demonstrated that PEI toxicity was related to the interaction of apoptosis-related proteins through PEI-protein interaction mechanism. Rahman et al. [74] found that the cytotoxicity of the materials was higher when the structure contained sulfur, long alkyl chain and aromatic ring. The high cytotoxicity resulting from the aromatic ring may be attributed to the strong π-π interaction between the aromatic ring and the cell membrane, resulting in rapid disruption of cellular Structure [75]. This resulted in high cytotoxicity of AM-zein-SA at concentrations greater than 62.5 mg/L.

### Statistical analysis

The statistical analyses were performed by one-way ANOVA using IBM SPSS Statistics 24.0 Version, and every experiment’s technical specifications were triplicated. Significant differences in means were compared using Duncan’s test at a significance level of p ≤ 0.05.

## Conclusions

Herein, the anti-UV property of the nano-pesticide was improved by grafting of SA onto AM-zein. The sustained-release experiment showed that the nano-pesticide carrier could achieve the sustained-release of the pesticide in 72 h, with good photostability and storage stability. Moreover, the foliar affinity of the carrier improved EB retention on leaves and the spreadability and adhesivity on the surface prevented the loss of pesticides due to photolysis and washing away with rain after spraying. The drug release behavior of EB@AM-zein-SA conformed to the first-order kinetic equation and the driving force of drug release was the difference in drug concentration. AM-zein-SA, without undergoing any changes in its insecticidal activity, could simultaneously improve the salt stress resistance and salt stress germination rate of cucumber, reduce growth inhibition due to stress under high-concentration salt, and had a good effect on crops. In addition, EB@AM-zein-SA obviously improved the upward transmission rate of EB, as compared with EB. In view of the rapid increase of the area of saline-alkali land caused by climate or environmental deterioration and the increasing demand for grain, it is necessary to develop more diversified and three-dimensional strategies to deal with the problem of grain production in saline-alkali land. In this study, SA grafted onto zein-based nano-pesticide carrier. The biodegradable nanomaterials have low toxicity at appropriate concentrations. It reduces the possibility of environmental or biological toxicity caused by the enrichment of nanoparticles through the food chain. Which provided a green strategy to control plant diseases, insects, and pests while reducing salt stress on crops in saline-alkali soil.

## Data Availability

All data generated or analysed during this study are included in this published article and its Additional information files.

## References

[1] Hayes TB, Hansen M (2017). From silent spring to silent night: agrochemicals and the anthropocene. Elem Sci Anth.

[2] Nuruzzaman MD, Rahman MM, Liu Y, Naidu R (2016). Nanoencapsulation, nano-guard for pesticides: a new window for safe application. J Agr Food Chem..

[3] Zhao D, Zhang P, Ge L, Zheng GJ, Wang X, Liu W, Yao Z (2018). The legacy of organochlorinated pesticides (OCPs), polycyclic aromatic hydrocarbons (PAHs) and polychlorinated biphenyls (PCBs) in Chinese coastal seawater monitored by semi-permeable membrane devices (SPMDs). Mar Pollut Bull.

[4] Pirsaheb M, Moradi N (2020). Sonochemical degradation of pesticides in aqueous solution: investigation on the influence of operating parameters and degradation pathway—a systematic review. Rsc Adv.

[5] Hao L, Lin G, Chen C, Zhou H, Chen H, Zhou X (2019). Phosphorylated zein as biodegradable and aqueous nanocarriers for pesticides with sustained-release and anti-UV properties. J Agr Food Chem.

[6] Kacsó T, Neaga IO, Erincz A, Astete CE, Sabliov CM, Oprean R, Bodoki E (2018). Perspectives in the design of zein-based polymeric delivery systems with programmed wear down for sustainable agricultural applications. Polym Degrad Stabil..

[7] Liu Y, Li S, Li H, Alomgir HM, Sameen DE, Dai J, Qin W, Lee K (2021). Synthesis and properties of core-shell thymol-loaded zein/shellac nanoparticles by coaxial electrospray as edible coatings. Mater Design..

[8] Yuan Y, Ma M, Xu Y, Wang D (2022). Surface coating of zein nanoparticles to improve the application of bioactive compounds: a review. Trends Food Sci Tech.

[9] Chen L, Lin Y, Zhou H, Hao L, Chen H, Zhou X (2021). A stable polyamine-modified zein-based nanoformulation with high foliar affinity and lowered toxicity for sustained avermectin release. Pest Manag Sci.

[10] Wang L, Ning C, Pan T, Cai K (2022). Role of Silica Nanoparticles in abiotic and biotic stress tolerance in plants: a review. Int J Mol Sci.

[11] Ku Y, Sintaha M, Cheung M, Lam H (2018). Plant hormone signaling crosstalks between biotic and abiotic stress responses. Int J Mol Sci.

[12] Ding Z, Kheir AM, Ali OA, Hafez EM, ElShamey EA, Zhou Z, Wang B, Ge Y, Fahmy AE, Seleiman MF (2021). A vermicompost and deep tillage system to improve saline-sodic soil quality and wheat productivity. J Environ Manage.

[13] Rajput VD, Singh RK, Verma KK, Sharma L, QuirozFigueroa FR, Meena M, Gour VS, Minkina T, Sushkova S, Mandzhieva S (2021). Recent developments in enzymatic antioxidant defence mechanism in plants with special reference to abiotic stress. Biology.

[14] Rajput VD, Minkina T, Kumari A, Singh VK, Verma KK, Mandzhieva S, Sushkova S, Srivastava S, Keswani C (2021). Coping with the challenges of abiotic stress in plants: new dimensions in the field application of nanoparticles. Plants.

[15] Singh A, Sengar RS, Rajput VD, Minkina T, Singh RK (2022). Zinc oxide nanoparticles improve salt tolerance in rice seedlings by improving physiological and biochemical indices. Agriculture.

[16] Xie W, Chen Q, Wu L, Yang H, Xu J, Zhang Y (2020). Coastal saline soil aggregate formation and salt distribution are affected by straw and nitrogen application: a 4-year field study. Soil Till Res..

[17] Ali Q, Sehrai GH, Hussain Z, Sajid M, Abbas G, Salisu IB, Shahid AA (2017). Genetically modified crops and their biosafety concerns. NY Sci J.

[18] Ashraf M, Munns R (2022). Evolution of approaches to increase the salt tolerance of crops. Crit Rev Plant Sci.

[19] Cai XZ, Zheng Z (1997). Biochemical mechanisms of salicylic acid—induced resistance rice seeding blast. Acta Phytopathol Sin.

[20] Wang YF, Xie L, He C, Ma C, Wu Y (2015). Effects of iduction time and concentration of exogenous SA and MeJA on phenolic compounds in *Gossypium spp* leaves. Acta Agric Boreali Occident Sin.

[21] Song WY, Peng SP, Shao CY, Shao HB, Yang HC (2014). Ethylene glycol tetra-acetic acid and salicylic acid improve anti-oxidative ability of maize seedling leaves under heavy-metal and polyethylene glycol 6000-simulated drought stress. Plant Biosyst.

[22] Wang Y, Zhang H, Hou P, Su X, Zhao P, Zhao H, Liu S (2014). Foliar-applied salicylic acid alleviates heat and high light stress induced photoinhibition in wheat during the grain filling stage by modulating the gene transcription and antioxidant defense. Plant Growth Regul.

[23] Zhang Q, Li D, Wang Q, Song X, Wang Y, Yang X, Qin D, Xie T, Yang D (2021). Exogenous salicylic acid improves chilling tolerance in maize seedlings by improving plant growth and physiological characteristics. Agronomy.

[24] Taherbahrani S, Zoufan P, Zargar B (2021). Modulation of the toxic effects of zinc oxide nanoparticles by exogenous salicylic acid pretreatment in *Chenopodium*
*murale*
*L.*. Environ Sci Pollut Res Int.

[25] Li T, Hu Y, Xuhua Du, Tang H, Shen C, Wu J (2014). Salicylic acid alleviates the adverse effects of salt stress in *Torreya*
*grandis* cv. Merrillii seedlings by activating photosynthesis and enhancing antioxidant systems. PLoS ONE.

[26] Pirasteh-Anosheh H, Emam Y, Pessarakli M (2019). Grain filling pattern of *Hordeum vulgare* as affected by salicylic acid and salt stress. J Plant Nutr.

[27] Mohammadi H, Rahimpour B, PirastehAnosheh H, Race M (2022). Salicylic acid manipulates ion accumulation and distribution in favor of salinity tolerance in *Chenopodium quinoa*. Int J Env Res Pub He.

[28] Larsen T, Fernández C (2017). Enzymatic-fluorometric analyses for glutamine, glutamate and free amino groups in protein-free plasma and milk. J Dairy Res.

[29] Huang JL, Wang XK (2015). Principles and techniques of plant physiological biochemical experiment.

[30] Bach-Pages M, Preston GM, Medina C, López-Baena FJ (2018). Methods to quantify biotic-induced stress in plants. Methods in molecular biology.

[31] Yan L, Li P, Zhao X, Ji R, Zhao L (2020). Physiological and metabolic responses of maize (*Zea mays*) plants to Fe_3_O_4_ nanoparticles. Sci Total Environ.

[32] Tomba JP, Silva LI, García Genga M, Barrera Galland G, Perez CJ (2019). Characterizing chemical composition of polyolefin-based copolymers from spectral features in the C-H stretching region. J Raman Spectrosc.

[33] Freeman JM, Henshall T (1968). Group vibrations and the vibrational analysis of molecules containing methylene groups. Part I. The basic equations and the application of the method to methylene dichloride and cyclopropane. Can J Chem.

[34] Abdolmohammad-Zadeh H, Salimi A (2020). A magnetic adsorbent based on salicylic acid-immobilized magnetite nano-particles for pre-concentration of Cd(II) ions. Front Chem Sci Eng.

[35] Xu C, Xie F, Guo X, Yang H (2005). Synthesis and cofluorescence of Eu(Y) complexes with salicylic acid and o-phenanthroline. Spectrochim Acta Part A Mol Biomol Spectrosc.

[36] Meng D, Zhou H, Xu J, Zhang S (2021). Studies on the interaction of salicylic acid and its monohydroxy substituted derivatives with bovine serum albumin. Chem Phys.

[37] Gillgren T, Barker SA, Belton PS, Georget DMR, Stading M (2009). Plasticization of zein: a thermomechanical, FTIR, and dielectric study. Biomacromol.

[38] Sadat A, Joye IJ (2020). Peak fitting applied to fourier transform infrared and raman spectroscopic analysis of proteins. Appl Sci.

[39] Karaca C, Atac A, Karabacak M (2015). Conformational analysis, spectroscopic study (FT-IR, FT-Raman, UV, ^1^H and ^13^C NMR), molecular orbital energy and NLO properties of 5-iodosalicylic acid. Spectrochim Acta Part A Mol Biomol Spectrosc.

[40] Lu YB, Yang P, Huang WN, Yang YN, Wu JZ (2010). 5-Formylsalicylic acid and 5-(benzimidazolium-2-yl)salicylate. Acta Crystallogr C.

[41] Zhang X, Dong C, Hu Y, Gao M, Luan G (2021). Zein as a structural protein in gluten-free systems: an overview. Food Sci Human Wellness.

[42] Zhang J, Zhou W, Xu M, Fang C, Du Q, Xu X, Lyu F, Ding Y, Liu J (2021). Characterization of silver carp myosin glycated with phosphorylated konjac oligo-glucomannan. J Sci Food Agr.

[43] Hood C, Laredo T, Marangoni AG, Pensini E (2021). Water-repellent films from corn protein and tomato cutin. J Appl Polym Sci.

[44] Zhao M, Li P, Zhou H, Hao L, Chen H, Zhou X (2022). pH/redox dual responsive from natural polymer-based nanoparticles for on-demand delivery of pesticides. Chem Eng J.

[45] Liu Z, Cao X, Ren S, Wang J, Zhang H (2019). Physicochemical characterization of a zein prepared using a novel aqueous extraction technology and tensile properties of the zein film. Ind Crops Prod.

[46] Nguyen KT, Tran PH, Ngo HV, Tran TT (2021). Hydrophobic and hydrophilic film-forming gels for the controlled delivery of drugs with different levels of hydrophobicity. Anticancer Agent Med Chem..

[47] Wang Q, Tang Y, Yang Y, Lei L, Lei X, Zhao J, Zhang Y, Li L, Wang Q, Ming J (2022). Interactions and structural properties of zein/ferulic acid: the effect of calcium chloride. Food Chem.

[48] Huang S, He J, Han L, Lin H, Liu G, Zhang W (2020). Zein-polyglycerol conjugates with enhanced water solubility and stabilization of high oil loading emulsion. J Agr Food Chem.

[49] Zou Y, Zhong J, Pan R, Wan Z, Guo J, Wang J, Yin S, Yang X (2017). Zein/tannic acid complex nanoparticles-stabilised emulsion as a novel delivery system for controlled release of curcumin. Int J Food Sci Technol.

[50] Schmidt G, Woods JT, Fung LXB, Gilpin CJ, Hamaker BR, Wilker JJ (2019). Strong adhesives from corn protein and tannic acid. Adv Sustain Syst.

[51] Zhang J, Yang J, Yang Y, Luo J, Zheng X, Wen C, Xu Y (2019). Transcription factor CsWIN1 regulates pericarp wax biosynthesis in cucumber grafted on pumpkin. Front Plant Sci.

[52] Shafiq S, Akram NA, Ashraf M, GarcíaCaparrós P, Ali OM, Latef AA (2021). Influence of glycine betaine (natural and synthetic) on growth, metabolism and yield production of drought-stressed maize (*Zea mays L.*) plants. Plants.

[53] Rajabi DA, Zahedi M, Ludwiczak A, Piernik A (2022). Foliar application of salicylic acid improves salt tolerance of sorghum (*Sorghum bicolor (L.) Moench*). Plants.

[54] Abedini M, Hassani BD (2015). Salicylic acid affects wheat cultivars antioxidant system under saline and non-saline condition. Russ J Plant Physiol.

[55] Desoky ES, Merwad AR, Abo El-Maati MF, Mansour E, Arnaout SM, Awad MF, Ramadan MF, Ibrahim SA (2021). Physiological and biochemical mechanisms of exogenously applied selenium for alleviating destructive impacts induced by salinity stress in bread wheat. Agronomy.

[56] Zamaninejad M, Khorasani SK, Moeini MJ, Heidarian AR (2013). Effect of salicylic acid on morphological characteristics, yield and yield components of Corn (*Zea mays L.*) under drought condition. Eur J Exp Biol.

[57] Vakilian H, Andres RE, Habibi RL, Behmanesh M (2020). Fabrication and optimization of linear PEI-modified crystal nanocellulose as an efficient non-viral vector for *In-Vitro* gene delivery. Rep Biochem Mol Biol.

[58] Qi C, Lin X, Li S, Liu L, Wang Z, Li Y, Bai R, Xie Q, Zhang N, Ren S, Zhao B (2019). SoHSC70 positively regulates thermotolerance by alleviating cell membrane damage, reducing ROS accumulation, and improving activities of antioxidant enzymes. Plant Sci.

[59] Liu Z, Ma C, Hou L, Wu X, Wang D, Zhang L, Liu P (2022). Exogenous SA affects rice seed germination under salt stress by regulating Na^+^/K^+^ balance and endogenous GAs and ABA homeostasis. Int J Mol Sci.

[60] Sundaria N, Singh M, Upreti P, Chauhan RP, Jaiswal JP, Kumar A (2019). Seed priming with iron oxide nanoparticles triggers iron acquisition and biofortification in wheat (*Triticum*
*aestivum* L.) grains. J Plant Growth Regul.

[61] Aydin A, Kurt F, Hürkan K (2021). Key aromatic amino acid players in soybean (*Glycine max*) genome under drought and salt stresses. Biocatal Agric Biotechnol.

[62] Bravo SA, Lamas MC, Salamón CJ (2002). *In-vitro* studies of diclofenac sodium controlled-release from biopolymeric hydrophilic matrices. J Pharm Pharm Sci.

[63] Alexa IF, Ignat M, Popovici RF, Timpu D, Popovici E (2012). *In vitro* controlled release of antihypertensive drugs intercalated into unmodified SBA-15 and MgO modified SBA-15 matrices. Int J Pharm.

[64] Ritger PL, Peppas NA (1987). A simple equation for description of solute release I. Fickian and non-fickian release from non-swellable devices in the form of slabs, spheres, cylinders or discs. J Control Release.

[65] Creed D (1984). The photophysics and photochemistry of the near-UV absorbing amino acids-II. Tyrosine and its simple derivatives. Photochem Photobiol.

[66] Wang Y, Padua GW (2012). Nanoscale characterization of zein self-assembly. Langmuir.

[67] Briggs GG, Bromilow RH, Evans AA, Williams M (1982). Relationships between lipophilicity and root uptake and translocation of non-ionised chemicals by barley. Pestic Sci.

[68] Ge J, Lu M, Wang D, Zhang Z, Liu X, Yu X (2016). Dissipation and distribution of chlorpyrifos in selected vegetables through foliage and root uptake. Chemosphere.

[69] Zhu H, Chen L, Xing W, Ran S, Wei Z, Amee M, Wassie M, Niu H, Tang D (2020). Phytohormones-induced senescence efficiently promotes the transport of cadmium from roots into shoots of plants: a novel strategy for strengthening of phytoremediation. J Hazard Mater.

[70] de Moura GF, de Souza PN, Campos FG, Mantoan LPB, Boaro CSF (2020). Exogenous salicylic acid modifies gas exchange and biomass production of *'Mentha x piperita' L*. Aust J Crop Sci.

[71] Homayoonzadeh M, Esmaeily M, Talebi K, Allahyari H, Nozari J, Michaud JP (2020). Micronutrient fertilization of greenhouse cucumbers mitigates pirimicarb resistance in *Aphis gossypii* (hemiptera: aphididae). J Econ Entomol.

[72] Hall A, Larsen AK, Parhamifar L, Meyle KD, Wu LP, Moghimi SM (2013). High resolution respirometry analysis of polyethylenimine-mediated mitochondrial energy crisis and cellular stress Mitochondrial proton leak and inhibition of the electron transport system. Biochim Biophy Acta..

[73] Khansarizadeh M, Mokhtarzadeh A, Rashedinia M, Taghdisi SM, Lari P, Abnous KH, Ramezani M (2016). Identification of possible cytotoxicity mechanism of polyethylenimine by proteomics analysis. Hum Exp Toxicol.

[74] Moshikur RM, Chowdhury MR, Wakabayashi R, Tahara Y, Moniruzzaman M, Goto M (2018). Characterization and cytotoxicity evaluation of biocompatible amino acid esters used to convert salicylic acid into ionic liquids. Int J Pharm.

[75] Hou XD, Liu QP, Smith TJ, Li N, Zong MH (2017). Evaluation of toxicity and biodegradability of cholinium amino acids ionic liquids. PLoS ONE.

